# Neuregulin signaling mediates the acute and sustained antidepressant effects of subanesthetic ketamine

**DOI:** 10.1038/s41398-021-01255-4

**Published:** 2021-02-24

**Authors:** Steven F. Grieco, Xin Qiao, Kevin G. Johnston, Lujia Chen, Renetta R. Nelson, Cary Lai, Todd C. Holmes, Xiangmin Xu

**Affiliations:** 1 Department of Anatomy and Neurobiology, School of Medicine, University of California, Irvine, CA 92697-1275 USA; 2 Department of Mathematics, University of California, Irvine, CA 92697-3875 USA; 3 Department of Psychological and Brain Sciences, Indiana University, Bloomington, IN 47405-7000 USA; 4 Department of Physiology and Biophysics, School of Medicine, Universityof California, Irvine, CA 92697- 4560 USA; 5 The Center for Neural Circuit Mapping, University of California, Irvine, CA 92697 USA; 6 Department of Biomedical Engineering, University of California, Irvine, CA 92697-2715 USA; 7 Department of Microbiology and Molecular Genetics, University of California, Irvine, CA 92697-4025 USA; 8 Department of Computer Science, University of California, Irvine, CA 92697-3435 USA

**Keywords:** Neuroscience, Physiology

## Abstract

Subanesthetic ketamine evokes rapid antidepressant effects in human patients that persist long past ketamine’s chemical half-life of ~2 h. Ketamine’s sustained antidepressant action may be due to modulation of cortical plasticity. We find that ketamine ameliorates depression-like behavior in the forced swim test in adult mice, and this depends on parvalbumin-expressing (PV) neuron-directed neuregulin-1 (NRG1)/ErbB4 signaling. Ketamine rapidly downregulates NRG1 expression in PV inhibitory neurons in mouse medial prefrontal cortex (mPFC) following a single low-dose ketamine treatment. This NRG1 downregulation in PV neurons co-tracks with the decreases in synaptic inhibition to mPFC excitatory neurons for up to a week. This results from reduced synaptic excitation to PV neurons, and is blocked by exogenous NRG1 as well as by PV targeted ErbB4 receptor knockout. Thus, we conceptualize that ketamine’s effects are mediated through rapid and sustained cortical disinhibition via PV-specific NRG1 signaling. Our findings reveal a novel neural plasticity-based mechanism for ketamine’s acute and long-lasting antidepressant effects.

## Introduction

One of the major limitations of the frontline drugs for treating clinical depression is their very slow onset of effectiveness, typically between 3 and 6 weeks^1^. Ketamine has gained widespread attention for its potential to treat psychiatric disorders at subanesthetic doses, particularly in human patients who are resistant to classical treatments for depression^2^. Though ketamine is considered to be an NMDA receptor antagonist at high doses, its mechanism of action at subanesthetic doses is unknown. Ketamine’s chemical half-life time is only ~2 h^3^, yet a single treatment results in antidepressant effects that last up to ~2 weeks, well past the chemical persistence of ketamine or its metabolites^4^. A potential explanation for these acute and long-lasting effects is that ketamine modulates neural circuit plasticity. This is supported conceptually by findings that behavioral depression results from a failure of neural plasticity^5^. Administration of antidepressants, such as fluoxetine and ketamine, induce cortical plasticity in adult animals^6,7^. But whether and how ketamine induces cortical plasticity in a rapid and sustained fashion is unknown.

Ketamine modulates the excitatory/inhibitory (E/I) balance in cortical circuits^8–10^. This change in E/I balance may initiate long-lasting cortical plasticity in higher-order association cortex such as prefrontal cortex (PFC). There are reductions in synapse function in the PFC of both depressed patients and rodents with depression-like behavior^8,10–12^. And substantial evidence shows that a subanesthetic dose of ketamine increases synaptic activity in mPFC^8,10,13,14^. Our previous work shows that genetic silencing of parvalbumin-expressing (PV) interneuron activity results in cortical disinhibition and promotes cortical plasticity beyond the visual development critical period^15,16^. Work from our group and others has indicated that neuregulin-1 (NRG1) signaling through PV neurons regulates developmental and adult visual cortical plasticity^16,17^. PV neurons have strong and concentrated NRG1 expression; this distinguishes them from surrounding putative excitatory neurons^16^. NRG1’s receptor ErbB4 is highly restricted to PV neurons^16,18–24^. Downregulation of NRG1/ErbB4 signaling by PV interneurons results in a rapid retraction of excitatory inputs to PV cells, resulting in cortical disinhibition, and the initiation of cortical plasticity^25^. Recently, we discovered that a subanesthetic dose of ketamine induces adult visual cortical plasticity by downregulating NRG1 signaling in PV inhibitory neurons in adult mice^26^. This motivated us to test the hypothesis that the antidepressant effects of a single subanesthetic ketamine treatment are due to its modulation of NRG1-directed signaling in PV inhibitory neurons in higher association cortex including PFC.

In the present study, we find that ketamine’s antidepressant effects, as measured in the forced swim test (FST), depend on PV neuron-directed NRG1/ErbB4 signaling. We find that a single dose of subanesthetic ketamine induces acute and sustained downregulation of NRG1 expression in PV inhibitory neurons in medial prefrontal cortex (mPFC), resulting in sustained PV excitatory input loss and cortical disinhibition. Our study establishes molecular, cellular, and circuit mechanisms for ketamine’s antidepressant actions.

## Results

### Antidepressant effects of ketamine depend on NRG1/ErbB4 signaling

To determine if the ketamine-mediated amelioration of depression-like behaviors depends on NRG1/ErbB4 signaling in PV inhibitory interneurons, we tested the antidepressant effects of ketamine in the FST using mice, by manipulating NRG1/ErbB4 signaling. The FST measures depressive-like behavioral states and strongly predicts antidepressant efficacy^27^. The reduction in depression-like behavior using the FST after a subanesthetic dose of ketamine is the most well-characterized behavioral measure of ketamine’s antidepressant effect to date in rodents^28^. The antidepressant effects of ketamine for the FST behavioral despair test are well established.

**Fig. 1:**
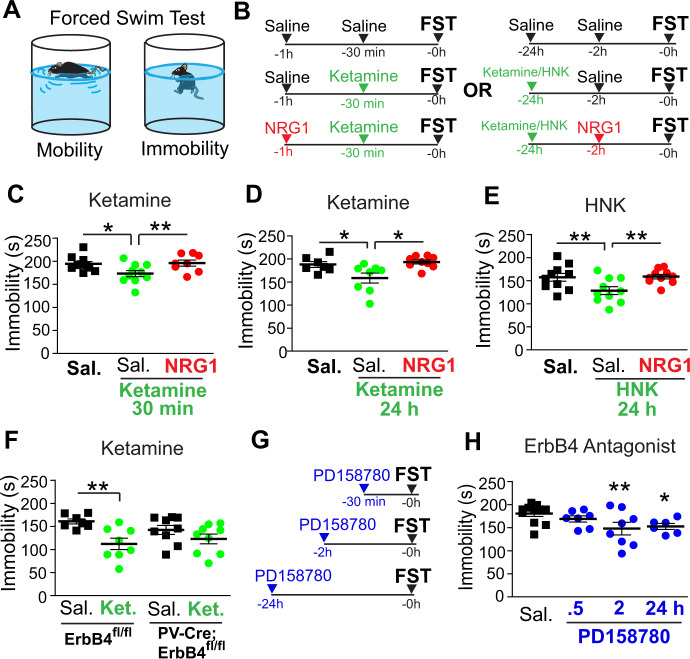
Antidepressant effects of subanesthetic ketamine depend on NRG1/ErbB4 signaling. **A** A schematic illustration of the forced swim test (FST) which was used to determine the dependence of the antidepressant effect of ketamine on NRG1/ErbB4 signaling. **B** Schematics for the experimental timelines (30 min or 24 h after treatment) for ketamine and HNK (left and right). **C** Wild-type mice were treated with saline (*n* = 7), ketamine (10 mg/kg; s.c.)(*n* = 8), or ketamine plus NRG1 (1 µg/mouse)(*n* = 9) and were then subjected to the FST 30 min later. Immobility times are shown (One-way ANOVA: overall *p* = 0.0069. Two-sample *t* test: ketamine + saline vs saline *p* = 0.0411, ketamine+NRG1 vs ketamine+saline *p* = 0.0066; mean ± SEM). **D** Wild-type mice were treated with saline (*n* = 10), ketamine (10 mg/kg; s.c.)(*n* = 10), or ketamine plus NRG1 (1 µg/mouse)(*n* = 10) and were subjected to the FST 24 hours later (One-way ANOVA: overall *p* = 0.0289. Two-sample *t* test: ketamine + saline vs saline *p* = 0.0257, ketamine + NRG1 vs ketamine+saline *p* = 0.0351; mean ± SEM). **E** Wild-type mice were treated with saline (*n* = 11), HNK (10 mg/kg; s.c.)(*n* = 14), or HNK plus NRG1 (1 µg/mouse)(*n* = 10) and were subjected to the FST 24 h later (One-way ANOVA: overall *p* = 0.0031. Two-sample *t* test: HNK + saline vs saline *p* = 0.0076, HNK + NRG1 vs HNK + saline *p* = 0.0071; mean ± SEM). **F** Control ErbB4^fl/fl^ mice were treated with saline (*n* = 7) or ketamine (10 mg/kg; s.c.)(*n* = 8) and PV-Cre; ErbB4^fl/fl^ mice were treated with saline (*n* = 9) or ketamine (10 mg/kg; s.c.)(*n* = 9) and tested on the FST 24 hours later (Two-way ANOVA: overall *p* = 0.0033. Two-sample *t* test: saline vs ketamine (ErbB4^fl/fl^) *p* = 0.0041, saline vs ketamine (PV-Cre; ErbB4^fl/fl^) *p* = n.s.; mean ± SEM). We find no significant effect of genotype. **G** Schematics for the experimental timelines for the ErbB4 antagonist PD158780. **H** Wild-type mice were treated with vehicle (*n* = 10) or PD158780 (10 mg/kg; s.c.) and subjected to the FST 30 min (*n* = 7), 2 h (*n* = 8) or 24 h (*n* = 6) later (One-way ANOVA: overall *p* = 0.0421. Two-sample *t* test: PD158780 vs saline, 30 min. p = n.s., 2 h *p* = 0.0098, 24 h *p* = 0.0395; mean ± SEM).

A single subanesthetic ketamine treatment (10 mg/kg, subcutaneous, s.c.) shows significant antidepressant effects for the FST as measured by increased duration of immobility at 30 min and 24 h after treatment (Fig. 1A–D). To determine if NRG1 signaling underlies ketamine’s antidepressant effects, animals were treated with exogenous NRG1 at a dose we have previously found effective in the context of modulating visual cortical plasticity^16^. We used recombinant NRG1 containing only the EGF core domain of NRG1-β1. This form of NRG1 has been shown previously to penetrate the blood–brain barrier and to functionally activate ErbB4 in the cortex^16,29^. Subcutaneous injection of NRG1 alone has no effects on performance in the FST^30^. Exogenous NRG1 (1 µg per mouse, s.c.) significantly blocks the antidepressant effect of ketamine in both the acute 30 min and the long-term 24 h ketamine treatment groups (Fig. 1A–D) (Fig. 1C. One-way ANOVA: overall *p* = 0.0069. Two-sample *t* test: ketamine + saline vs saline *p* = 0.0411, ketamine + NRG1 vs ketamine + saline *p* = 0.0066) (Fig. 1D. One-way ANOVA: overall p = 0.0289. Two-sample *t* test: ketamine+saline vs saline *p* = 0.0257, ketamine + NRG1 vs ketamine + saline *p* = 0.0351). We then tested the effects of a single treatment with (2R, 6R)-hydroxynorketamine (HNK) (10 mg/kg, s.c.), the major metabolite of ketamine which has previously been shown to mediate ketamine’s effects and which does not appear to have abuse potential^9^. HNK shows significant antidepressant effects for the FST performed 24 h after treatment, and HNK’s effect is blocked by NRG1 treatment (Fig. 1E. One-way ANOVA: overall *p* = 0.0031. Two-sample *t* test: HNK + saline vs saline *p* = 0.0076, HNK + NRG1 vs HNK + saline *p* = 0.0071).

Since exogenous NRG1 treatment prevents ketamine-induced antidepressant effects, we tested specifically if NRG1/ErbB4 signaling by PV interneurons is required for the antidepressant effect of ketamine in the FST. This was done using PV-Cre; ErbB4^fl/fl^ mice in which ErbB4 expression is selectively removed in PV-positive interneurons^31^. The absence of ErbB4 in PV interneurons prevents ketamine’s antidepressant effects in the FST, whereas in control mice ketamine is still effective (Fig. 1F. Two-way ANOVA: overall *p* = 0.0033. Two-sample *t* test: saline vs ketamine (ErbB4^fl/fl^) *p* = 0.0041, saline vs ketamine (PV-Cre; ErbB4^fl/fl^) *p* = n.s.). We further explored the antidepressant dependence of ketamine action on ErbB4 signaling using the pharmacological ErbB antagonist PD158780, which acutely blocks ErbB4 signaling^17^. FST immobility significantly improves 2 hours and 24 hours following PD158780 treatment (Fig. 1H, One-way ANOVA: overall *p* = 0.0421. Two-sample *t* test: PD158780 vs saline, 30 min. *p* = n.s., 2 h *p* = 0.0098, 24 h *p* = 0.0395). These results are consistent with previous reports of the antidepressant effect of ketamine, and provide new evidence that NRG1/ErbB4 signaling in PV interneurons contributes to ketamine’s antidepressant actions.

### Ketamine increases neural activity of mPFC excitatory neurons in vivo

**Fig. 2:**
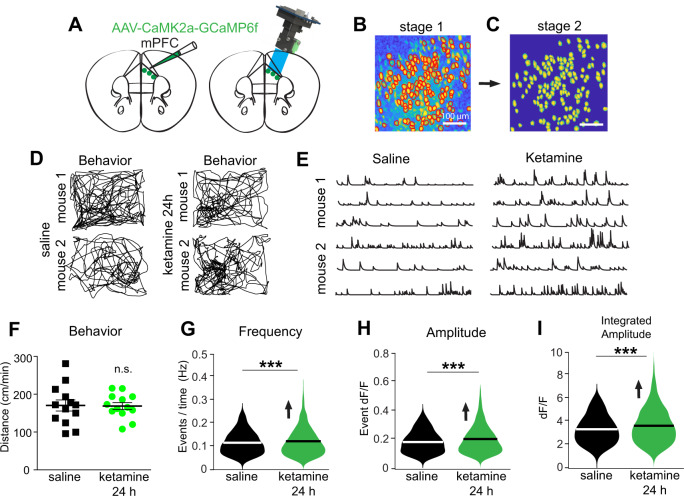
Subanesthetic ketamine increases mPFC excitatory neuron activities in vivo. **A** (Left) A schematic illustration of AAV injection for targeted expression of GCaMP6f in mPFC excitatory neurons. (Right) A schematic illustration of a miniaturized fluorescent microscope (miniscope) used to image in vivo calcium signals in mPFC excitatory neurons in awake and freely behaving mice. The GRIN lens, implanted over mPFC at 2 weeks after an AAV-CaMK2a-GCaMP6f injection into mPFC, is shown in blue. **B**, **C** Representative neuron footprints to be used for data analysis after stage 1 (CNMF-E)(B) and stage 2 (SCOUT) processing of the same data. **D** Behavioral tracking data (black lines) of two representative mice. The mice were introduced to a 35 cm × 25 cm behavioral arena under low-lighting conditions (<20 lux), and miniscope and behavioral recordings were made at baseline and 24 h after ketamine treatment (10 mg/kg; s.c.). **E** Calcium transient event data from three representative mPFC neurons from two different mice at baseline and 24 h after ketamine treatment. **F** There is no significant difference in locomotor behavior (normalized distance; cm/min) 24 h after ketamine treatment. **G** Frequencies, as determined by events per time (see “Methods” for details), for neurons at baseline and 24 h after ketamine (*n* = 1260 cells) (linear mixed effect model: overall, *p* = 3.66 × 10^−5^; violin plot with median). **H** Average peak amplitudes for neurons at baseline and 24 h after ketamine (*n* = 1260 cells) (linear mixed effect model: overall, *p* = 0.0003; violin plot with median). **I** Average integrated amplitudes for neurons at baseline and 24 h after ketamine (*n* = 1260 cells) (linear mixed effect model: overall, *p* = 0.0001; violin plot with median). Note that we performed multiple time-point imaging from control mice, neural activity levels did not differ at 1 h versus 24 h post saline injection.

To determine the effect of ketamine on cortical activity, we performed population calcium imaging of excitatory neurons in vivo in mPFC using head-mounted miniscopes^32–34^. We selectively expressed GCaMP6f in mPFC excitatory neurons by using stereotaxic guided injection of AAV-CaMK2a-GCaMP6f into mouse mPFC (Fig. 2A). We longitudinally tracked and measured the activity of mPFC excitatory neurons. We first probed baseline population calcium activities at single cell resolution for mPFC excitatory neurons, then in the same neurons we measured the effects of ketamine 24 h after subcutaneous administration (Fig. 2B, C). No differences in locomotion behavior are observed in mice 24 h after ketamine treatment relative to baseline (Fig. 2D, F). We pooled 1332 neurons from 12 mice, and found that compared to the baseline condition, ~95% of these neurons exhibited increased activity 24 h following ketamine treatment (Fig. 2E). The in vivo calcium event frequencies of these excitatory neurons in mPFC significantly increase (Fig. 2G. linear mixed effect model: overall, *p* = 3.66 × 10^−5^). Both calcium event peak amplitudes and integrated calcium event amplitudes in mPFC excitatory neurons show significant increases as well at 24 h following the ketamine treatment (Fig. 2H. linear mixed effect model: overall, *p* = 0.0003) (Fig. 2I: linear mixed effect model: overall, *p* = 0.0001). These in vivo imaging results support the hypothesis that ketamine administration results in cortical disinhibition in mPFC.

### Sustained cortical disinhibition evoked by subanesthetic ketamine

**Fig. 3:**
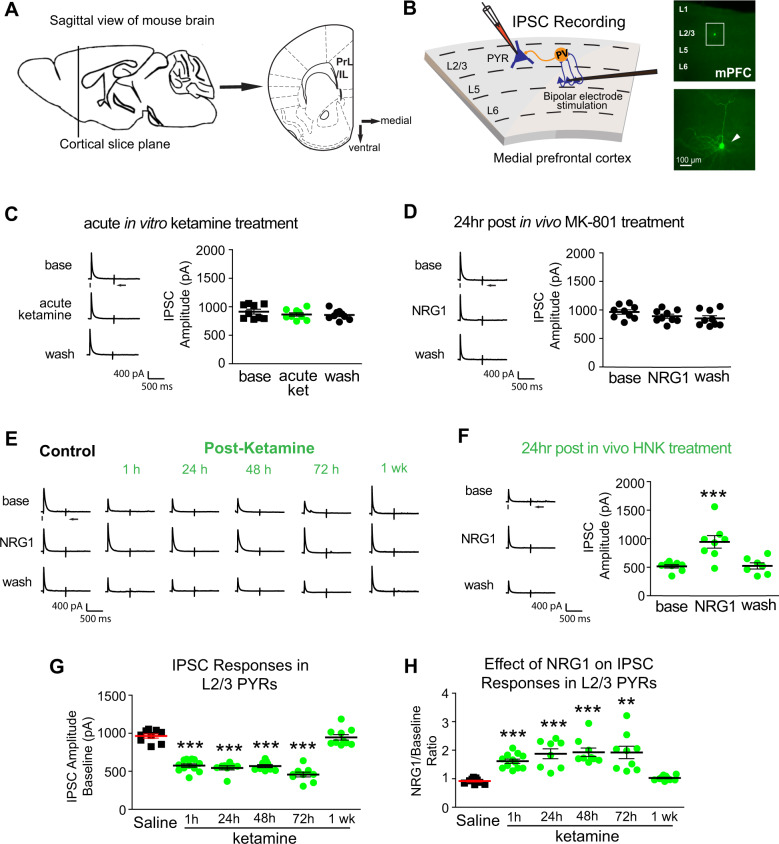
Subanesthetic ketamine evokes sustained cortical disinhibition, which is reversed with NRG1 treatment. **A** Schematic depicting the medial prefrontal cortex (mPFC) region of mouse brain from which recordings were taken. **B** Schematic of recording inhibitory postsynaptic currents (IPSCs) in L2/3 pyramidal (PYR) neurons by preferentially activating L5 → L2/3 feedforward projections to L2/3 PV neurons through L5 electrical stimulation. Recorded pyramidal neurons are filled with biocytin for post-hoc morphological confirmation. **C** Ketamine acutely applied to cortical slice baths does not induce any change in inhibitory inputs to L2/3 pyramidal neurons (*n* = 9 cells). Ketamine was washed into cortical slices at a bath concentration of 100 µM starting at 15 min before recording, and continuing through recording. **D** MK-801 in vivo treatment 24 h prior to recording does not induce any change in inhibitory inputs to L2/3 pyramidal neurons (*n* = 9 cells). MK-801 is injected to animals at a concentration of 0.1 mg/kg 24 h before recording. **E** However, ketamine in vivo treatment (10 mg/kg; s.c.) dramatically reduces evoked IPSCs to PYR neurons with acute and sustained long-term effects at 1 h (*n* = 12 cells), 24 h (*n* = 8 cells), 48 h (*n* = 9 cells), 72 h (*n* = 9 cells), and 1 week (*n* = 9 cells) following treatment. Ketamine-evoked sustained decreases in IPSCs are acutely reversed with bath application of NRG1 (5 nM). **F** HNK in vivo treatment (10 mg/kg; s.c.) 24 h before recording, reduces synaptic inhibition to L2/3 pyramidal cells. Bath application of NRG1 (5 nM) then reverses the effect of in vivo HNK treatment and increases inhibitory inputs (*n* = 8 cells) (Linear mixed effect model: overall, *p* = 2.09 × 10^−5^; NRG1 vs base *p* = 1.92 × 10^−5^, NRG1 vs wash *p* = 5.3 × 10^−5^; mean ± SEM). **G** Summary data of average evoked IPSC amplitudes in L2/3 pyramidal neurons under the specified conditions (saline, 1, 24, 48, and 72 h, and 1 week after ketamine treatment) (One-way ANOVA: overall *p* = 1.77 × 10^−20^. Two-sample *t* test (adjusted for multiple comparisons): in vivo ketamine vs saline, 1 h *p* = 5.01 × 10^−9^, 24 h *p* = 8.54 × 10^−8^, 48 h *p* = 1.69 × 10^−8^, 72 h *p* = 1.04 × 10^−8^, 1 week *p* = n.s.; mean ± SEM). **H** Acute NRG1 treatment increases average evoked IPSC amplitudes ratios in L2/3 pyramidal neurons under the specified conditions (control saline, 1, 24, 48, and 72 h, and 1 week after ketamine treatment) (One-way ANOVA: overall *p* = 2.4 × 10^−8^. Two-sample *t* test (adjusted for multiple comparisons): in vivo ketamine vs saline, 1 h *p* = 1.02 × 10^−6^, 24 h *p* = 1.81×10^−4^, 48 h *p* = 2.52 × 10^−5^, 72 h *p* = 0.0015, 1wk *p* = n.s.; mean ± SEM). For each trial, electrical stimulation (1 ms, 20 µA) was applied, represented by a black tick beneath one example trace. For the example trace, the arrow indicates the current injection response to monitor access resistance during the experiment.

Recent evidence suggests ketamine may induce cortical disinhibition by reducing interneuron activity^35,36^. To test the effect of ketamine on cortical inhibition in mPFC (Fig. 3A), we performed whole-cell recording of electrically evoked inhibitory postsynaptic currents (IPSCs) in L2/3 excitatory pyramidal neurons. We did this by preferentially activating L5 to L2/3 feedforward projections to L2/3 inhibitory neurons through L5 electrical stimulation (Fig. 3B). To determine if NMDAR inhibition mediates ketamine’s effects, we tested both ketamine acutely applied to the slice bath, or an in vivo treatment of the NMDAR antagonist MK-801. Neither bath-applied ketamine nor an MK-801 treatment caused appreciable effects on IPSC amplitudes in L2/3 pyramidal neurons (Fig. 3C, D). This suggests that it is unlikely that acute NMDAR inhibition significantly modulates inhibitory synaptic inhibition to L2/3 excitatory neurons in mPFC.

Since it is proposed that ketamine undergoes metabolism in vivo and its metabolites may mediate ketamine’s antidepressant effect, we tested the disinhibitory action of ketamine by injecting mice with subanesthetic ketamine 1 h before preparing mPFC slices for IPSC recording. We find dramatic reductions in evoked IPSC amplitudes in mPFC excitatory cells at 1 h after ketamine treatment, when inhibitory input to L2/3 excitatory cells is ~50% of saline-treated controls (Fig. 3E, G, H). We also tested the effect of the major ketamine metabolite HNK on cortical disinhibition; HNK decreases pyramidal IPSCs 24 h after treatment (Fig. 3F). Previously, we found that bath application of exogenous NRG1 increases excitatory cell IPSCs under conditions in which the cortex has downregulated NRG1 signaling^16^. We then tested if increased NRG1 signaling reverses the disinhibitory effects. Bath application of NRG1 (5 nM) reverses the disinhibitory effects of ketamine and HNK, and increases evoked IPSCs in L2/3 pyramidal neurons treated with in vivo ketamine and HNK (Fig. 3E, F, H).

Next we examined the sustained actions of ketamine treatment on evoked IPSCs in L2/3 pyramidal neurons in mPFC slices recorded at 24, 48, 72 h and 1 week after a single-dose treatment. The inhibitory input to excitatory pyramidal neurons is significantly reduced at these time points after ketamine treatment (Fig. 3E, G, H). Evoked IPSCs then return to baseline levels 1 week after ketamine treatment (Fig. 3G. One-way ANOVA: overall *p* = 1.77 × 10^−20^. Two-sample *t* test (adjusted for multiple comparisons): in vivo ketamine vs saline, 1 h *p* = 5.01 × 10^−9^, 24 h *p* = 8.54 × 10^−8^, 48 h *p* = 1.69 × 10^−8^, 72 h *p* = 1.04 × 10^−8^, 1 week *p* = n.s.). Ketamine-mediated decreases in evoked IPSCs are acutely reversible with bath application of NRG1 (Fig. 3E, G, H). NRG1 effects are quickly eliminated with washout, and NRG1 has no effect on IPSC amplitude in normal control pyramidal neurons that have not been treated with ketamine (Fig. 3H. One-way ANOVA: overall *p* = 2.4 × 10^−8^. Two-sample *t* test (adjusted for multiple comparisons): in vivo ketamine vs saline, 1 h *p* = 1.02 × 10^−6^, 24 h *p* = 1.81 × 10^−4^, 48 h *p* = 2.52 × 10^−5^, 72 h *p* = 0.0015, 1 week *p* = n.s.). Neither ketamine treatment nor NRG1 has a significant effect on spontaneous IPSCs or paired-pulse ratios (Supplementary Fig. 1). These findings support that NRG1 signaling underlies the disinhibitory effects of ketamine in the mPFC.

### NRG1/ErbB4 signaling in PV neurons reduced by ketamine

**Fig. 4:**
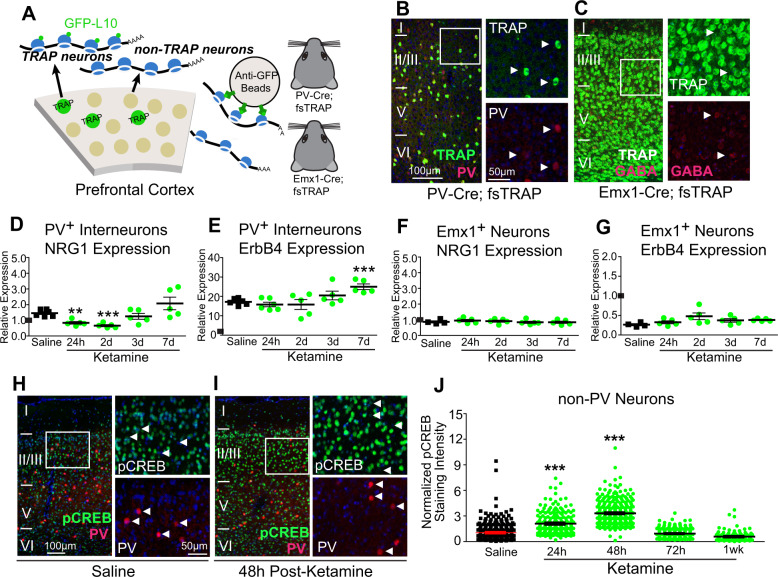
NRG1 expression in PV neurons is reduced by ketamine. **A**–**G** Testing the effect of ketamine on NRG1 mRNA expression using TRAP. **A** A schematic illustration of the translating ribosome affinity purification strategy^37^. Using PV-Cre; fsTRAP mice, translating ribosomes (polysomes) from PV cells (green cells) have EGFP tags from the EGFP-L10 transgene. Lysis of all cells in the PV-Cre; fsTRAP cortex releases both tagged and non-tagged polysomes. The tagged polysomes are selectively captured on an anti-GFP affinity matrix and used for purification of PV-specific mRNAs associated with tagged polysomes. This strategy is also used to purify mRNA from Emx1+ neurons using Emx1-Cre; fsTRAP mice. Mice were treated with saline or ketamine at P56, then sacrificed 24, 48, 72 h or 1 week later for fsTRAP extraction and qPCR analysis. See “Methods” for more information. **B** Confocal images of genetically labeled PV cells (green), PV immunolabeling (red) and their overlay in layer I-VI of mouse mPFC in P56 PV-Cre; fsTRAP-EGFP mice; Scale bar = 100 µm. The white boxes indicate the region of mPFC imaged at higher magnification (**B**, right); Scale bar = 50 µm. **C** Confocal images of genetically labeled excitatory cells (green), GABA immunolabeling (red) and their overlay in layer I-VI of mouse mPFC in P56 Emx1-Cre; fsTRAP-EGFP mice. The white boxes indicate the region of mPFC imaged at higher magnification (**C**, right). Arrowheads indicate GABA immunopositive cells. **D**–**G** All results were analyzed using the ddCt method with *gapdh* as an endogenous control, along with normalization to Emx1+ P56 *nrg1* expression, as indicated by a black mark on the *y*-axis, so that all panels (**D**–**G)** can be compared^71^. (**D**) Ketamine treatment (10 mg/kg; s.c.) significantly lowers NRG1 mRNA expression levels from PV neurons (PV-Cre; fsTRAP) in mPFC in P56 mice (*n* = 5–6 samples; each sample is pooled from 5 mice for PV cell-specific data) (One-way ANOVA: overall *p* = 0.0006. Two-sample *t* test (adjusted for multiple comparisons): ketamine vs saline, 24 h (1d) *p* = 0.0027, 48 h (2d) *p* = 0.0002; mean ± SEM). **E** PV cell-specific mRNA expression levels of ErbB4 from mice treated with saline or ketamine (One-way ANOVA: overall *p* = 0.0018. Two-sample *t* test (adjusted for multiple comparisons): ketamine vs saline, 1 week *p* = 4.55 × 10^−4^); mean ± SEM. **F** Emx1+ cell-specific (Emx1-Cre; fsTRAP) NRG1 mRNA expression levels from mPFC cortices (*n* = 4–5 samples; each sample is pooled from two mice for Emx1+ cell-specific data) is not significantly changed with ketamine treatment (10 mg/kg; s.c.). **G** Emx1+ cell-specific ErbB4 mRNA expression levels are not significantly changed with ketamine treatment (10 mg/kg; s.c.), and are much lower than in PV cells. **H**–**J** Testing the effect of ketamine on pCREB expression in excitatory neurons. **H** Confocal images of genetically labeled PV cells (red), pCREB immunolabeling (green) and their overlay in layer I-VI of mouse mPFC in P56 PV-Cre; Ai9 mice treated with saline; Scale bar = 100 µm. The white box indicates the region of mPFC digitally enlarged (**H**, right); Scale bar = 50 µm. White arrowheads indicate that PV neurons have very low pCREB immunoreactivity. **I** Confocal images of genetically labeled PV cells (red), pCREB immunolabeling (green) and their overlay in layer I-VI of mouse mPFC in P56 PV-Cre; Ai9 mice 48 h after ketamine treatment (10 mg/kg; s.c.). The white boxes indicate the region of mPFC digitally enlarged (I, right). White arrowheads indicate that PV neurons have very low pCREB immunoreactivity. **J** Quantification of the increase in pCREB immunoreactivity in mPFC 24 and 48 h after ketamine treatment. After 72 h to 1 week pCREB levels return to baseline (linear mixed effect model: overall *p* = 4.3 × 10^−32^, ketamine vs saline, 24 h *p* = 2.37 × 10^−5^, 48 h p = 1.51 × 10^−23^; mean ± SEM). Please see the “Methods” section for quantification and normalization of immunostaining intensity; the overall normalized values from different mice were compared across treatment groups (*n* = 6 for saline; *n* = 3–6 for ketamine groups).

Next, we asked whether ketamine modulates NRG1/ErbB4 signaling specifically in PV interneurons. We tested this using translating ribosome affinity purification (TRAP), which allows for the measurement of cell type-specific mRNA expression changes^37^ (Fig. 4A). NRG1 and ErbB4 mRNA expression levels were measured from PV+ or Emx1+ neurons that were either PV+ or non-GABA+, respectively (Fig. 4B, C). Then tissue from mPFC was harvested from mice at 24 h (1 day), 48 h (2 days), 72 h (3 days) or 1 week after ketamine treatment. Expression of NRG1 mRNA in PV cells of control, non-treated mouse mPFC is higher than that of Emx1+ neurons (Fig. 4D, F). In PV interneurons, but not in Emx1+ neurons, ketamine treatment results in a sustained downregulation of NRG1 mRNA expression which returns to baseline levels only after 3 days (Fig. 4D, F) (Fig. 4D. One-way ANOVA: overall *p* = 0.0006. Two-sample *t* test (adjusted for multiple comparisons): ketamine vs saline, 24 h *p* = 0.0027, 48 h *p* = 0.0002). In PV neurons ErbB4 expression is markedly higher than in Emx1+ neurons. ErbB4 mRNA expression does not significantly change in either PV or Emx1+ neurons after ketamine treatment, except for the 1 week time-point for PV cells where ErbB4 was increased (Fig. 4E, G) (Fig. 4E. One-way ANOVA: overall *p* = 0.0018. Two-sample *t* test (adjusted for multiple comparisons): ketamine vs saline, 1week *p* = 4.55 × 10^−4^).

We then investigated whether ketamine-mediated downregulation of NRG1 mRNA expression in PV neurons in mPFC was associated with increases in molecular correlates of enhanced neural activity and cortical disinhibition. Using immunostaining we measured the levels of phosphorylated cAMP response element transcription factor (pCREB) at 24, 48, 72 h or 1 week after ketamine treatment (Fig. 4H–J). In response to increased neural activity in excitatory neurons, CREB is phosophorylated at Ser133 (pCREB) resulting in the transcription of genes induced by neural activity^38^. We quantified the levels of pCREB staining in L2/3 of mPFC in ketamine-treated mice. In excitatory neurons pCREB levels are significantly increased and this is sustained for up to 2 days after ketamine treatment (Fig. 4J.Linear mixed effect model: overall *p* = 4.3 × 10^−32^, ketamine vs saline, 24 h *p* = 2.37 × 10^−5^, 48 h = 1.51 × 10^−23^). The levels of pCREB then returned to baseline levels. PV interneuronal pCREB levels at baseline are very low and are not significantly changed with ketamine treatment. These findings suggest that a single subanesthetic ketamine treatment results in decreased PV NRG1/ErbB4-directed signaling and cortical disinhibition as measured by pCREB immunoreactivity in excitatory neurons.

### PV excitatory input loss evoked by ketamine

**Fig. 5:**
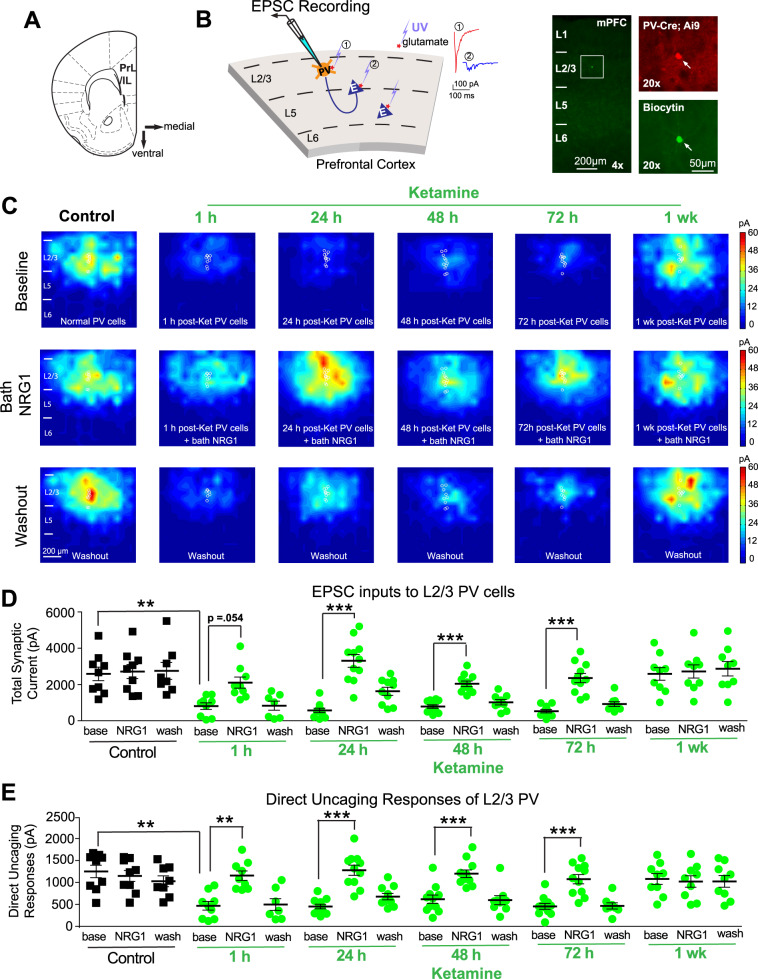
Ketamine induces a sustained loss of excitatory inputs to PV neurons, which is restored with NRG1. **A** Schematic depicting the medial prefrontal cortex (mPFC) region of mouse brain from which recordings were taken. **B** Schematic of laser-scanning photostimulation (LSPS) mapping of cortical synaptic connections to individually recorded PV neurons in mPFC slices. LSPS maps the broad spatial pattern of synaptic inputs to the neuron of interest, and distinguishes direct uncaging responses (1, red) to assess glutamate-mediated excitability/responsiveness at perisomatic locations, and synaptically mediated EPSC responses (2, cyan) to assess synaptic inputs from presynaptic neuronal spiking. Cortical layers of 1, 2/3, 5, and 6 in the brain slice are indicated as L1, L2/3, L5, and L6. Recorded PV neurons are filled with biocytin for post-hoc confirmation. **C** Group-averaged, excitatory input maps of PV cells recorded at the specified conditions. White circles in each map represent individual PV neurons. The color scales code integrated excitatory input strength (blue = low, red = high) and applies to all other maps in the same condition. **C** (left column) An acute bath application of NRG1 (5 nM) does not significantly modulate local excitatory synaptic inputs onto PV neurons in mPFC of saline-treatment control mice. Averaged excitatory input maps of L2/3 PV cells are shown before (top), during bath NRG1 (20 min after NRG1 application) (middle), and after washout of bath NRG1 (bottom). **C** (right columns) Reduced excitatory inputs to PV neurons are seen at 1, 24, 48, and 72 h after ketamine treatment (10 mg/kg; s.c.), but not after 1 week. Excitatory inputs to PV neurons in ketamine-treated mice are restored by acute bath application of NRG1. This acute restoration by NRG1 is eliminated by washout of the bath NRG1. **D** Summary data of average total synaptic input strength measured for L2/3 PV neurons under the specified conditions (control, 1, 24, 48, and 72 h and 1 week after ketamine treatment) (Linear mixed effects model (two factors): highly significant. Two-sample *t* test (adjusted for multiple comparisons): base vs base (control), 1 h *p* = 0.0057, 24 h *p* = 2.76 × 10^−4^, 48 h *p* = 7.54 × 10^−4^, 72 h *p* = 1.42 × ^10-4^, 1 week *p* = n.s. Paired sample *t* test (adjusted for multiple comparisons): NRG1 vs base, 1 h *p* = 0.054, 24 h *p* = 7.23 × 10^−5^, 48 h *p* = 2.38 × 10^−4^, 72 h *p* = 1.82 × 10^−4^, 1week *p* = n.s.; mean ± SEM). **E** Direct uncaging responses before and during bath NRG1 for PV neurons in control versus ketamine-treated mice. Peak direct responses were measured, which are not affected by overriding synaptic inputs (Linear mixed effects model (two factors): highly significant; Two-sample *t* test (adjusted for multiple comparisons): base vs base (control), 1 h *p* = 0.0032, 24 h *p* = 1.96 × 10^−4^, 48 h *p* = 0.012, 72 h *p* = 3.77 × 10^−4^, 1wk *p* = n.s.; Paired sample *t* test (adjusted for multiple comparisons): NRG1 vs base, 1 h *p* = 0.0095, 24 h *p* = 2.63 × 10^−4^, 48 h *p* = 7.95 × 10^−4^, 72 h *p* = 2.92 × 10^−4^, 1 week *p* = n.s.; mean ± SEM).

In order to determine if excitatory synaptic inputs to PV interneurons are the functional locus regulating ketamine-mediated cortical disinhibition in mPFC, the effects of ketamine treatment on excitatory inputs to PV cells were measured. We mapped the spatial extent and strengths of excitatory input to L2/3 PV interneurons in mPFC using laser-scanning photostimulation (LSPS) in mPFC brain slices^15,39,40^ (Fig. 5A, B). The LSPS experimental method is effective for mapping local circuit connections (Supplementary Fig. 2). It involves first recording from a single neuron (L2/3 PV interneuron), and then spatially restricted UV laser stimulating surrounding cortical sites in a sequential manner in order to evoke action potentials (APs) with glutamate release (Fig. 5B). Recording from the postsynaptic neuron allows us to quantitatively assess the pattern and strength of synaptic inputs to the recorded neuron from photostimulated sites. Experiments were performed in mPFC slices prepared from control mice and in vivo ketamine-treated adult mice at 1, 24, 48, 72 h, and 1 week after treatment. Control PV neurons were found to have robust excitatory inputs from L2/3 and L5 (Fig. 5C). In mice treated with ketamine, the excitatory drive to PV interneurons is dramatically reduced at 1, 24, 48, and 72 h after treatment (Fig. 5C, top row, D, E). At 1 week post ketamine treatment, the excitatory inputs to PV neuronsare restored to control levels (Fig. 5C–E). Therefore, excitatory input to PV interneurons following ketamine treatment is highly reduced, and this effect is sustained for ~1 week.

Next we examined if enhancing NRG1 signaling would restore the excitatory input to PV interneurons after ketamine treatment. We first performed baseline recordings, and then applied bath NRG1 to cortical slices for 20 min. Bath NRG1 had no effect on control PV cells that were not treated with ketamine. Neither the local excitatory synaptic input currents nor the direct uncaging responses (as measured by responses to uncaging at perisomatic regions) were altered in non-ketamine-treated control PV cells after NRG1 application (Fig. 5C–E). In contrast to this, both the excitatory synaptic currents and the direct uncaging responses of PV cells are very strongly enhanced by bath application of NRG1 in ketamine-treated mice at the 1, 24, 48, and 72 h time points (Fig. 5C–E) (Fig. 5C, D. Linear mixed effects model (two factors: treatment, time): highly significant. Two-sample *t* test (adjusted for multiple comparisons): base vs base (control), 1 h *p* = 0.0057, 24 h *p* = 2.76 × 10^−4^, 48 h *p* = 7.54 × 10^−4^, 72 h *p* = 1.42 × 10^−4^, 1 week *p* = n.s.; Paired sample *t* test (adjusted for multiple comparisons): NRG1 vs base, 1 h *p* = 0.054, 24 h *p* = 7.23 × 10^−5^, 48 h *p* = 2.38 × 10^−4^, 72 h *p* = 1.82 × 10^−4^, 1week *p* = n.s.) (Fig. 5C, E. Linear mixed effects model (two factors): highly significant; Two-sample *t* test (adjusted for multiple comparisons): base vs base (control), 1 h *p* = 0.0032, 24 h *p* = 1.96 × 10^−4^, 48 h *p* = 0.012, 72 h *p* = 3.77 × 10^−4^, 1 week *p* = n.s. Paired sample *t* test (adjusted for multiple comparisons): NRG1 vs base, 1 h *p* = 0.0095, 24 h *p* = 2.63 × 10^−4^, 48 h *p* = 7.95 × 10^−4^, 72 h *p* = 2.92 × 10^−4^, 1 week *p* = n.s.). Bath-applied NRG1 does not alter resting membrane potential or intrinsic membrane excitability in PV cells under control conditions or following ketamine treatment (Supplementary Fig. 3). These findings from the circuit mapping experiments allow us to determine the molecular and circuit locus of ketamine-mediated disinhibition in the mPFC (Supplementary Fig. 4).

## Discussion

Our study defines a novel and critical role of NRG1/ErbB4 signaling in mPFC PV inhibitory interneurons in mediating the acute and sustained antidepressant effects of subanesthetic ketamine. The most commonly used antidepressant medications, including selective serotonin reuptake inhibitors, tricyclic antidepressants, monoamine oxidase inhibitors, and lithium show 3-6 weeks of time lags to reach effective management of clinical depression^1^. The time lag for antidepressant effectiveness is critical because of the risk of suicide in depressed patients. In contrast, ketamine shows both rapid and sustained antidepressant effects^4^. We find that ketamine-induced antidepressant effects are induced by the downregulation of PV-specific NRG1 signaling and the concurrent loss of excitatory inputs to PV cells. We do not yet fully understand the detailed molecular mechanisms for how/why NRG1 downregulation in PV interneurons occurs after ketamine administration. This is a topic for future investigation. It is possible that a specific NRG1 isoform is responsible for our observed effects^41^. Ketamine’s effects are blocked by exogenous NRG1 or knockout of its receptor ErbB4 in PV cells. Our findings are supported by the proposal that ketamine’s antidepressant effects are mediated by its regulation of neural circuit plasticity^5^. A single treatment of ketamine or its metabolite HNK induces molecular and circuit changes that promote neural plasticity that outlasts the physical presence of these therapeutic reagents.

Our work adds to and integrates previous studies toward understanding the mechanism of how ketamine mediates both rapid and sustained antidepressant effects. Ketamine induces BDNF production^3^, which can signal through its receptor TrkB, resulting in activation of mTOR, all of which are implicated in neural plasticity^8,10,42^. mTOR signaling then inhibits GSK3, which also regulates neural plasticity and is the target of the mood stabilizer lithium^43^. All of these signaling molecules are regulated by ketamine treatment^44^. However, it is not clear what circuit mechanisms induce these molecular events. It is becoming increasingly apparent that ketamine treatment results in cortical disinhibition^35,36,45,46^. One potential mechanism for the disinhibitory action of ketamine and the induction of plasticity-related molecular events would be ketamine-mediated decreases in interneuronal activity.

In this study, we have extended the concept of PV NRG1/ErbB4 signaling, cortical disinhibition and plasticity, which we and others have formulated in the visual cortex^16,17,26^, to the mPFC, which is relevant for cognition and mood disorders. NRG1 signaling is critical for maintaining excitatory synaptic inputs onto PV cells, while reductions in PV NRG1/ErbB4 signaling results in cortical disinhibition^16,17^. We have previously established that exogenous NRG1 rapidly restores excitatory inputs onto deprived PV cells through downstream PKC-dependent activation and AMPA receptor exocytosis^16^. This mechanism is important for cortical plasticity not only in juvenile mice, but in adult mice as well. As PV cells are the most prominent interneuron type in cortex, and as ErbB4 is very highly expressed and localized in PV cells^47^, we believe that this mechanism is highly significant. While the present study focuses on mPFC mechanisms, systemic ketamine treatment has global effects on multiple brain regions, including lateral habenula, and other brain areas that likely also contribute to its antidepressant effects^48,49^.

Our data indicate a neural mechanism of ketamine-mediated cortical disinhibition that is independent of acute NMDAR antagonism, as neither bath-applied ketamine itself nor MK-801 modulates synaptic inhibition (IPSCs) to L2/3 pyramidal neurons. This is consistent with the proposed NMDAR inhibition-independent mechanism of ketamine’s actions^9^. While the IPSC evoked by stimulating layer 5 may involve other types of inhibitory neurons, this does not affect our interpretation of cortical disinhibition. We also find that the ketamine metabolite HNK has NRG1-dependent circuit and behavioral effects. Previous studies have demonstrated that acute NRG1 subcutaneous injection alone in mice has no antidepressant-like effects while longer treatment regimens have measurable effects; depressed patients show a disruption of NRG1 signaling^30,50^. There has been criticism of the use of the FST to measure depression-like behavior in rodents^51^. We find that subanesthetic ketamine reduces PV NRG1/ErbB4 signaling, and that this co-tracks a reduction in excitatory input to PV cells, resulting in sustained cortical disinhibition in mPFC. While our data show a reduction of excitatory input to PV neurons, we have not directly examined inhibitory input to PV cells. However, we reason that the overall ketamine effect is to reduce PV neuron activity as reflected by cortical disinhibition in vivo and in vitro (Figs. 2 and 3). Using in vivo 2-photon calcium imaging, we have previously demonstrated a reduction of in vivo PV neuron activity in visual cortex.

Ketamine-mediated antidepressant effects in the FST depend on PV NRG1/ErbB4 signaling. Our new findings present a critical role for PV NRG1/ErbB4 signaling in the antidepressant effect of subanesthetic ketamine treatment, providing a compelling molecular and circuit mechanism for ketamine-mediated cortical disinhibition and plasticity (Supplementary Fig. 4). Taken together, we establish a PV inhibitory neuron-directed molecular and circuit mechanism to account for ketamine’s rapid and sustained effects on cortical plasticity that contributes to a better understanding of depression and related affective disorders.

## Methods

### Animals

All experimental procedures and protocols were approved by the Institutional Animal Care and Use Committee of the University of California, Irvine. To enable PV-specific labeling and mRNA expression analysis, PV-IRES-Cre knock-in (Jackson Laboratory, stock #008069) mice were crossed to fsTRAP mice^37^(Jackson Laboratory, stock #022367) to generate PV-Cre^+/−^; fsTRAP mice, in which translating polyribosomes of PV cells are tagged with EGFP from the GFP-L10 transgene. To enable excitatory neuron labeling and mRNA expression analysis, Emx1-Cre mice were crossed to fsTRAP mice to generate Emx1-Cre^+/−^; fsTRAP mice. Emx1 is predominantly expressed in cortical excitatory neurons^52^. For all experiments, mice were hemizygous for both transgenes. To genetically label PV cells PV-Cre mice were crossed with Ai9 tdTomato reporter knock-in mice (Jackson Laboratory, stock #007905). To generate ErbB4 conditional knockout mice, mice homozygous for loxP-flanked alleles^31^ were crossed with PV-Cre mice to produce PV-Cre^+/-^; ErbB4^flx/+^ mice. These mice were then crossed back to the homozygous loxP-flanked ErbB4 mice to produce PV-Cre^+/-^; ErbB4^flx/flx^ mice. The animals (2–5 mice per cage) were housed in a vivarium room with a 12-h light/dark cycle with access to food and water ad libitum. All behavioral experiments were performed between 9 and 12 a.m.

Please see Supplementary Table 1 for detailed experimental and animal information. The mice were randomly assigned to groups with treatment of either saline or subanesthetic ketamine. A single treatment of ketamine (10 mg/kg, s.c., ketamine hydrochloride; VedCo., Inc.) was used for all the experiments. The (2R, 6R)-HNK dosage (10 mg/kg; Tocris Bioscience) of the published study^9^ was used for our in vivo HNK treatment. For some experiments, we performed in vivo exogenous NRG1 treatment (1 µg per mouse) via subcutaneous administration of recombinant NRG1 containing only the EGF core domain of NRG1-β1 (R&D systems). This form of NRG1 has been shown previously to penetrate the blood–brain barrier and functionally activate ErbB4 in the cortex^29^. For PD158780 (Tocris) experiments, mice were either injected with drug (10 mg/kg; s.c.) or with vehicle (s.c.) containing 10% Tween 80, 20% DMSO, and 70% saline before testing.

### Forced swim test

For the FST, mice were habituated in the behavior room for 1 h before testing. Mice were then placed individually in a transparent glass cylinder containing water (24 cm high, 14.5 cm diameter, 14 cm water depth) at 23–25 °C and forced to swim. Mice were videotaped for 6 min, and the immobility time (time spent passively floating) was recorded for the last 4 min, after discarding activity in the first 2 min during which an animal tries to escape and habituates to the cylinder. ANY-MAZE software was used to record and analyze immobility (Stoelting Co.). Manual scoring of immobility was also confirmed by a blind experimenter. Immobility times were yielded by subtracting mobility time from the 4 min total testing time.

### Miniscope imaging experiments

At 2 weeks after AAV1-CaMKII-GCaMP6f injection, a gradient refractive index (GRIN) lens was implanted at the injection site in the mPFC as previously described by ref. ^34^. A circular craniotomy was centered at the coordinates for the mPFC injections site. ACSF was repeatedly applied to the exposed tissue, and the dura was carefully removed. Cortex below the craniotomy was aspirated. The GRIN lens (0.25 pitch, 0.55 numerical aperture, 1.8-mm diameter and 4.31 mm in length, Edmund Optics) was slowly lowered at an oblique angle (Fig. 2A) using a stereotaxic arm to mPFC. Next, a skull screw was used to anchor the GRIN lens to the skull. Both the GRIN lens and skull screw were fixed with cyanoacrylate and dental cement. Kwik-Sil (World Precision Instruments) was used to cover the lens. Two weeks later, a small aluminum baseplate was cemented onto the head of the animal atop the existing dental cement. A miniscope was fitted into the baseplate and locked in a position so that the field of view was in focus to visualize GCaMP6f-expressing neurons and visible landmarks, such as blood vessels.

For behavioral experiments involving mPFC, mice were first handled for 10 min per day for a week in order to allow the animals to become comfortable with the behavioral experimenter. Then animals were habituated to the behavioral arena for 10 min per day for 2 days without a miniscope. Behavior was performed in a quiet designated behavioral room under low lighting conditions (<20 lux). The behavioral arena was 35 cm × 25 cm in size with bedding which was changed between animals. Animals were then habituated to the behavioral arena with the miniscope fixed onto their head while they behaved freely for 10 min per day for 3 days, so that animals were acclimated and habituated to the behavioral experimenter, arena, and miniscope gear. After preparations were completed, in vivo GCaMP6-based calcium imaging of population mPFC neurons was performed in awake freely behaving mice. Behavioral recording were made by tracking the location of the LED on the miniscope through the experimental sessions. Sessions were 5–10 min in length. Using the behavioral tracking video data, the position and speed of the animal was determined using a custom Matlab script.

Please refer to previous publications^33,53^ and www.miniscope.org/ for technical details of our custom-constructed miniscopes. The head-mounted scope has a mass of about 3 g and uses a single, flexible coaxial cable to carry power, control signals, and imaging data to custom open-source data acquisition (DAQ) hardware and software. Under our experimental conditions, the miniscope has a 700 μm × 450 μm field of view with a resolution of 752 pixels × 480 pixels (~1 μm per pixel). The electronics packaged the data to comply with the USB video class protocol and then transmitted the data over SuperSpeed USB to a PC running custom DAQ software. The DAQ software was written in C++ and uses Open Computer Vision libraries for image acquisition. Images were acquired at ~30 frames/s and recorded to uncompressed.avi files. The DAQ software simultaneously records the behavior of an animal through a high-definition webcam (Logitech) at ~30 frames/s, with time stamping of both video streams for offline alignment.

Miniscope videos were first concatenated and downsampled by a factor of two using NIH ImageJ software, and then motion-corrected using the NoRMCorre MATLAB package^54^. Next, we visually inspected the maximum intensity projection images of individual video sessions and manually performed minor linear translations to align videos. Then, we created a large combined dataset by concatenating all aligned video images. While this large data concatenation required significant computer resources (that is, 28 cores, 128 GB dynamic random-access memory, 1 TB solid state drive, 8 TB hard disk drive), it greatly enhanced our ability to obtain cell tracking from different days. Subsequent analysis was performed using custom Matlab scripts. We adopted the newly developed method of extended constrained non-negative matrix factorization for endoscopic data (CNMF-E)^55^ to extract the calcium activity of individual neurons. The CNMF-E is based on the CNMF framework^56^, which enables simultaneously denoising, deconvolving and demixing of calcium imaging data. Its key features include modeling the large rapidly fluctuating background that has a low spatial-frequency structure and allows good separation of single-neuron signals from this background. After iteratively solving a constrained matrix factorization problem, CNMF-E extracts the spatial footprints of neurons and their associated temporal calcium signal traces. Specifically, the first step of estimating the temporal activity of a neuron (neuron.C is a denoised version of dF, which is the change of the fluorescence intensity over time) is computing the weighted average of fluorescence intensities after subtracting the temporal activity of other neurons within the region of interest of that neuron. Then, a deconvolution algorithm, OASIS^57^, was applied to obtain the deconvolved calcium event activity (neuron.S). To reduce the number of false discoveries, a noted problem with CNMF-E, we used SCOUT^58^, which applies a linear classifier to the proposed pixel intensity spatial footprints for each neuron, at each iteration. Proposed footprints were normalized and compared with a family of predefined probability distributions, believed to accurately describe typical neuron footprints. Amplitudes were calculated as follows: (1) For each cell, the peak values of calcium events that exceed 0.1*max (calcium peak values) were found, (2) the peak values (neuron.S (dF)) were measured. Integrated amplitudes were calculated as the summation of the integrated area under the calcium event signal (neuron.C (dF)).

### Translating ribosome affinity purification (TRAP)

Purification of polysomally bound mRNA from prefrontal cortical lysate was performed as described with modifications^37^. Briefly, mPFC was dissected in ice-cold ACSF (in mM: 126 NaCl, 2.5 KCl, 26 NaHCO_3_, 2 CaCl_2_, 2 MgCl_2_, 1.25 NaH_2_PO_4_, and 10 glucose). The entire mPFC region was dissected out from both hemispheres of each mouse, using a brain block and scalpel. Pooled cortex from 2 to 5 mice was grinded to powder on dry ice, followed with sonication for 5 s in ice-cold lysis buffer [20 mM HEPES (pH 7.4), 150 mM KCl, 5 mM MgCl_2_, 0.5 mM dithiothreitol, 100 μg/ml cycloheximide (Sigma-Aldrich), protease inhibitors (Roche) and 40 U/mL recombinant RNase inhibitor (Promega, Madison, WI)]. Homogenates were centrifuged for 10 min at 2000 × *g*, 4 °C, to pellet nuclei and large cell debris, and NP-40 (Invitrogen, Carlsbad, CA) and DHPC (Avanti Polar Lipids, Alabaster, Alabama) were added to the supernatant at final concentrations of 1% (vol/vol) and 30 mM, respectively. After incubation on ice for 5 min, the lysate was centrifuged for 10 min at 20,000 × *g* to pellet insoluble material. Three hundred microliters of the streptavidin Myone T1 dynabeads was bound to 120 μg biotinylated protein L first, then followed with two mouse monoclonal anti-GFP antibodies incubation (Htz-GFP19C8 and Htz-GFP19F7 (50 μg each, Memorial Sloan-Kettering Monoclonal Antibody Facility, New York, NY)). After being washed twice with the polysome extraction buffer, the beads were then added to the cell-lysate supernatant, and the mixture was incubated at 4 °C with end-over-end rotation overnight. The beads were subsequently collected on a magnetic rack and washed four times with high-salt polysome wash buffer [20 mM HEPES (pH 7.4), 350 mM KCl, 5 mM MgCl_2_, 1% NP-40, 0.5 mM dithiothreitol and 100 μg/mL cycloheximide]. RNA was eluted from the beads by incubating beads in RLT buffer (RNeasy Micro Kit, Qiagen, Venlo, Netherlands) with β-mercaptoethanol (10 μL/mL) for 5 min at room temperature. Eluted RNA was purified using RNeasy Micro Kit (Qiagen) per the manufacturer’s instructions including in-column DNase digestion. Immunoprecipitated RNA yield for each sample was ~20 ng/μL.

For PV-Cre; fsTRAP experiments, both cortices (mPFC) from 5 mice were pooled together to generate *n* = 1 before mRNA extraction. For Emx1-Cre; fsTRAP experiments both cortices (mPFC) from two mice were pooled together to generate *n* = 1 before mRNA extraction.

### Quantitative Real-Time Polymerse Chain Reaction (qPCR)

Purified RNA (30 ng) was converted to cDNA using Superscript^®^ III reverse transcriptase (Thermo Fisher Scientific, Waltham, MA, USA) according to the manufacturer’s instructions. Quantitative changes in cDNA levels were determined by real-time PCR using the Power SYBR Green Master Mix (Thermo Fisher Scientific, Waltham, MA, USA), using primers at a concentration of 500 nM. Primers were custom designed for mouse NRG1, ErbB4, and for the endogenous control GAPDH. Primers for NRG1 are specific for the “total” form, which represents all known transcript variants. PCR was carried out for 2 min 50 °C, 5 min 95 °C, 40 cycles (15 s for 95 °C, 30 s for 50 °C), followed by a melt curve. Technical triplicates were used. Technical triplicates were averaged together to produce a single Ct value for each gene of each sample. GAPDH was used to normalize gene expression and data presented at mean ± SEM. Cycling and quantitation were performed on a ViiA™ 7 Real-Time PCR System instrument (Thermo Fisher Scientific, Waltham, MA, USA) using the ViiA 7 software v1.2. The following primers were used: NRG1-F GCAAGTGCCCAAATGAGTTTAC; NRG1-R GCTCCTCCGCTTCCATAAAT; ErbB4-F CATGGCCTTCCAACATGACTCTGG; ErbB4-R GGCAGTGATTTTCTGTGGGTCCC; GAPDH-F CATCACTGCCACCCAGAAGACTG ; GAPDH-R ATGCCAGTGAGCTTCCCGTTCAG.

### Immunohistochemistry

For immunochemical staining experiments, animals were first deeply anesthetized with Uthasol (sodium pentobarbital, 100 mg/kg, i.p.) and were then perfused transcardially with 5 mL of 1× phosphate buffered saline (PBS, pH 7.3–7.4), followed by 20 mL 1× PBS containing 4% paraformaldehyde (PFA) and phosphatase inhibitor (PhosSTOP, 1 tablet for 20 mL, Roche, Switzerland). Brains were removed and maintained in 4% PFA for 24 h, and then transferred to 30% sucrose in 1× PBS for 24 h. Then, using a freezing microtome (Leica SM2010R, Germany), coronal sections of the brain were taken at a 30 µm thickness. Mouse mPFC coronal sections were used for immunohistochemical staining and analysis.

Free floating sections were rinsed five times with 1× PBS, and incubated in a blocking solution for 1 h at room temperature on a shaker. The blocking solution contained 5% normal donkey serum and 0.25% Triton X in 1× PBS. Sections were then incubated with the primary antibody diluted in blocking solution for 36 h at 4 °C. After incubation with the primary antibody, brain sections were rinsed thoroughly with 1× PBS, and then incubated with the secondary antibody diluted in blocking solution for 2 h at room temperature. After the secondary antibody was rinsed off, sections were counterstained with 10 µM 4′-6-diamidino-2- phenylindole (DAPI; Sigma-Aldrich, St. Louis, MO) for 5 min to help distinguish cortical laminar structure and neuronal nuclei. Lastly, sections were rinsed and then mounted on microscope slides. Sections were cover-slipped with Vectashield mounting medium (H-1000, Vector, Burlingame, CA). To identify PV-positive neurons in PV-Cre; fsTRAP mice, the primary goat anti-PV antibody (PVG-213, Swant, Switzerland; RRID:AB_10000345; 1:1000) and a Cy3-conjugated donkey anti-goat antibody (Jackson ImmunoResearch, 1:200) were used. To identify GABA positive neurons in Emx1-Cre; fsTRAP mice, the primary rabbit anti-GABA antibody (Sigma-Aldrich, 1:1000) and a Cy3-conjugated donkey anti-rabbit antibody (Jackson ImmunoResearch, 1:200) were used. To identify pCREB positive neurons, the primary rabbit anti-pCREB antibody (Cell Signaling, 1:1000) was used.

Immunostained sections were examined, and 10× and 40× image stacks were acquired using a confocal microscope (LSM 780, Carl Zeiss Microscopy, Germany). Image tiles, overlaying, maximum projections, and subset z-stack selections were performed using the Zeiss image processing software (ZEN). For fluorescent imaging, all sections of a staining series were acquired using the same settings (laser power, pinhole size, line scans), and data images were digitally processed identically.

Individual cell fluorescence measurements (immunofluorescence) are performed in final output images using Adobe Photoshop software (CS4 extended version, Adobe Systems, San Jose, CA). PV or putative excitatory neurons with clear somata in mPFC regions are selected; the cell areas (number of pixels) and their integrated density (whole-cell fluorescence intensity) are measured. The background reading of the fluorescence level is determined for each stained mPFC section image. The corrected total fluorescence per cell in arbitrary units is calculated in an Excel sheet by applying the measurements obtained from the analyzed cell with the formula: Corrected total cell fluorescence = Integrated density − (Area of selected cell × mean fluorescence of background reading)^59^. For immunostaining fluorescence quantification, the staining intensities (measured by corrected total fluorescence) of PV and non-PV neurons from individual mice (1–2 sections per mouse) in each group are quantified; the mean values with all examined neurons per each mouse are calculated. These mean values are normalized to the specified mean for the control condition in the same staining series. The overall normalized values from different mice were compared across different conditions.

### Electrophysiology and laser-scanning photostimulation

Coronal sections (400 µm thick) of mPFC were cut from mouse with a vibratome (VT1200S, Leica Biosystems, Buffalo Grove, IL) in sucrose-containing ACSF (85 mM NaCl, 75 mM sucrose, 2.5 mM KCl, 25 mM glucose, 1.25 mM NaH_2_PO_4_, 4 mM MgCl_2_, 0.5 mM CaCl_2_, and 24 mM NaHCO_3_). Slices were incubated for at least 30 min in sucrose-containing ACSF at 32 °C before being transferred into slice-recording chambers with standard ACSF (126 mM NaCl, 2.5 mM KCl, 26 mM NaHCO_3_, 2 mM CaCl_2_, 2 mM MgCl_2_, 1.25 mM NaH_2_PO_4_, and 10 mM glucose). Throughout the cutting, incubation and recording, the solutions were continuously supplied with 95% O_2_ to 5% CO_2_.

We have previously described our methods for electrophysiological recording, imaging, and photostimulation in detail, including the definitions of all reported parameters^40,60^. For our more recent publications using these same methods see refs. ^15,39,61,62^. Briefly, whole-cell recordings were performed in oxygenated ACSF at room temperature under a differential interference contrast/fluorescent Olympus microscope (BX51WI). Oxygenated ACSF was fed into the slice-recording chamber through a custom-designed flow system, driven by pressurized 95% O_2_ to 5% CO_2_ (3 PSI) with a perfusion flow rate of about 2 mL/min. Slices were first carefully examined under a 4× objective for targeting either L2/3 PV interneurons that express red fluorescent protein or tdTomato or pyramidal neurons within the mPFC. To perform whole-cell recordings, neurons were visualized at high magnification (60× objective, 0.9 NA; LUMPlanFl/IR, Olympus America Inc). The cell bodies of recorded neurons were at least 50 µm below the surface of the slice. Patch pipettes (4–6 MΩ resistance) made of borosilicate glass were filled with an internal solution containing 126 mM K-gluconate, 4 mM KCl, 10 mM HEPES, 4 mM ATP-Mg, 0.3 mM GTP-Na, and 10 mM phosphocreatine (pH 7.2, 300 mOsm) when measuring excitatory postsynaptic currents (EPSCs) at −70 mV. The electrodes also contained 0.1% biocytin for post-hoc cell labeling and further morphological labeling. Once stable whole-cell recordings were achieved with good access resistance (<30 MΩ), basic electrophysiological properties were examined through depolarizing and hyperpolarizing current injections. Electrophysiological data were acquired with a Multiclamp 700B amplifier (Molecular Devices), data acquisition boards (models PCI MIO 16E-4 and 6713, National Instruments), and a custom-modified version of Ephus software 5. Data were digitized at 10 kHz. Any recordings in which the access resistance changed by >20% during the course of the experiment were excluded from analysis. In separate experiments, a cesium-based internal solution containing 130 mM CsOH, 130 mM d-gluconic acid, 0.2 mM EGTA, 2 mM MgCl_2_, 6 mM CsCl, 10 mM HEPES, 2.5 mM ATP-Na, 0.5 mM GTP-Na, and 10 mM phosphocreatine (pH 7.2, 300 mOsm) was used to voltage clamp pyramidal neurons at the excitatory reversal potential (0–5 mV) and measure IPSCs. Electrically evoked inhibitory postsynaptic currents (IPSCs) in L2/3 excitatory pyramidal neurons were recorded by preferentially activating L5 to L2/3 feedforward projections to L2/3 inhibitory neurons through L5 electrical stimulation (Fig. 3).

During photostimulation experiments, the microscope objective was switched from 60× to 4× for laser-scanning photostimulation. The same low-power objective lens was used for delivering ultraviolet flash stimuli. Stock solution of MNI-caged-l-glutamate (Tocris Bioscience) was added to 20 mL of circulating ACSF for a concentration of 0.2 mM caged glutamate. The cortical slice image, acquired through the 4× objective, was visualized using a high-resolution digital CCD camera, and this was in turn was used for guiding and registering photostimulation sites. A laser unit (DPSS Lasers) was used to generate 355 nm UV laser pulses for glutamate uncaging. Short pulses of laser flashes (1 ms, 20 mW) were delivered using an electro-optical modulator and a mechanical shutter. Focal laser spots approximated a Gaussian profile with a diameter of ~50–100 μm.

Voltage clamping the recorded neuron allowed determination of sites contributing direct synaptic input. By systematically surveying synaptic inputs from hundreds of different sites across a large region, aggregate synaptic input maps were generated for individual neurons. For our mapping experiments, a standard stimulus grid (16 × 16 stimulation sites, 65 µm^2^ spacing) was used to tessellate mPFC from pia to white matter. The LSPS site spacing was empirically determined to separate adjacent stimulation sites by the smallest predicted distance in which photostimulation differentially activated adjacent neurons. Glutamate uncaging laser pulses were delivered sequentially in a nonraster, nonrandom sequence, following a “shifting-X” pattern designed to avoid revisiting the vicinity of recently stimulated sites^63^. Because glutamate uncaging agnostically activates both excitatory and inhibitory neurons, we empirically determined the excitatory and inhibitory reversal potentials in L2/3 pyramidal cells to properly isolate EPSCs and IPSCs. We voltage clamped PV and pyramidal cells at −70 mV to determine LSPS evoked EPSCs.

Photostimulation induces two kinds of excitatory responses: (1) responses that result from direct activation of the recorded neuron’s glutamate receptors, and (2) synaptically mediated responses (EPSCs) resulting from the suprathreshold activation of presynaptic excitatory neurons. Responses that occur within 10 ms of the laser pulse onset are considered direct; these responses exhibit a distinct waveform and occur immediately after glutamate uncaging. Synaptic currents with such short latencies are not possible because they would have to occur before the generation of APs in photostimulated neurons. Therefore, direct responses are excluded from local synaptic input analysis, but they are used to assess glutamate-mediated excitability/responsiveness of recorded neurons. At some locations, synaptic responses override the relatively small direct responses, and these responses are identified and included in synaptic input analysis. For data map analysis, we implement the approach for detection and extraction of photostimulation-evoked postsynaptic current responses described in ref. ^40^. LSPS evoked EPSCs are quantified across the 16 × 16 mapping grid for each cell, and 2–4 individual maps are averaged per recorded cell, reducing the likelihood of incorporating noise events in the analysis window. The EPSC input from each stimulation site is the measurement of the sum of individual EPSCs within the analysis window (>10 ms to 160 ms post photostimulation), with the baseline spontaneous response subtracted from the photostimulation response of the same site. The value is normalized with the duration of the analysis window (i.e., 150 ms) and expressed as average integrated amplitudes in picoamperes. The analysis window is used because photostimulated neurons fire most of their APs during this time. For the color-coded map displays, data are plotted as the average integrated EPSCs amplitude per pixel location (stimulation site), with the color scale coding input strength. For the group maps obtained across multiple cells, the individual cell maps were first aligned by their slice images using laminar cytoarchitectonic landmarks. Then a new map grid is created to re-sample and average input strength at each site location across cell maps; a smooth version of color-coded map is presented for overall assessments. To further quantitatively compare input strength across cell groups or different conditions, we measure the total ESPC inputs across all map sites (total synaptic input strength) for individual cells.

For the experiments that examined the effects of bath application of NRG1, MK-801, ketamine or HNK, the reagent(s) were added into the ACSF solution with the specified concentrations. The drug application for 20 min was estimated to produce full effects, while the washout of 20–30 min was considered to remove the added drug from the recording solution.

### Statistical analysis

Data analysis was conducted using Matlab and R. Because the data presented in this article were collected using different experimental designs, several appropriate statistical tests were applied in the data analysis. This included ANOVA, t-test, Kruskal–Wallis test, Friedman’s test, Mann–Whitney *U* test, Wilcoxon signed rank test, linear mixed effect model, and Bonferroni correction for multiple comparisons.

#### Linear mixed effect (LME) model

The LME model is a commonly used method to deal with repeated measures^64–67^. Its importance has been increasingly recognized in analyzing calcium imaging data^10,68–70^. We use LME in the presence of repeated measures. For example, the 1535 cells (observations) presented in Fig. 4J were from 24 mice. To account for random effects due to mice, we fit a linear mixed effects model where the random effect variable is mouse IDs. More complicated examples are the analyses we conducted for Figs. 2G–I. Take Fig. 2G as an example, 1332 cells were taken from 12 mice and each cell was measured twice, one at baseline and the other after ketamine treatment. Because there are repeated measures for each cell and cells are nested in mice, we fit an LME with nested random effects.

#### Normality and nonparametric tests

When normality does not hold or sample sizes are small, parametric results might not be accurate. For the results reported in the paper, we verified them by using nonparametric tests. Specifically, we substituted Kruskal–Wallis test for one-way ANOVA, Mann–Whitney *U* test for two-sample *t* test, Wilcoxon signed rank test for paired *t* test, Friedman’s test and its modified version for repeated measures one-way ANOVA. In most situations, the *p* values based on nonparametric tests are greater than those from parametric tests but the trends and conclusions remain valid.

#### Multiple comparisons

When there is more than one hypothesis test, appropriate adjustments are necessary to gauge claimed type I error rates and thereby to generate replicable research findings. The Bonferroni correction, which multiplies each *p* value by the number of tests considered, is a simple and stringent method to control familywise type I error rate. When the number of tests in a figure is a concern (e.g., more than two tests), we correct *p* values using the Bonferroni’s correction.

#### Software

The analysis was done using custom-written scripts in Matlab and R.

## Supplementary information

Supplementary Table 1

Supplementary Figure 1

Supplementary Figure 2

Supplementary Figure 3

Supplementary Figure 4

Supplementary Figure Legends

## Data Availability

The data that support the findings of this study are available from the corresponding author upon reasonable request.

## References

[1] Perlman K (2019). A systematic meta-review of predictors of antidepressant treatment outcome in major depressive disorder. J. Affect Disord..

[2] Berman RM (2000). Antidepressant effects of ketamine in depressed patients. Biol. Psychiatry.

[3] Autry AE (2011). NMDA receptor blockade at rest triggers rapid behavioural antidepressant responses. Nature.

[4] Zarate CA (2006). A randomized trial of an N-methyl-D-aspartate antagonist in treatment-resistant major depression. Arch. Gen. Psychiatry.

[5] Price RB, Duman R (2020). Neuroplasticity in cognitive and psychological mechanisms of depression: an integrative model. Mol. Psychiatry.

[6] Maya Vetencourt JF (2008). The antidepressant fluoxetine restores plasticity in the adult visual cortex. Science.

[7] Castren E, Rantamaki T (2010). The role of BDNF and its receptors in depression and antidepressant drug action: reactivation of developmental plasticity. Dev. Neurobiol..

[8] Li N (2010). mTOR-dependent synapse formation underlies the rapid antidepressant effects of NMDA antagonists. Science.

[9] Zanos P (2016). NMDAR inhibition-independent antidepressant actions of ketamine metabolites. Nature.

[10] Moda-Sava, R. N. et al. Sustained rescue of prefrontal circuit dysfunction by antidepressant-induced spine formation. *Science* **364**, 10.1126/science.aat8078 (2019). 10.1126/science.aat8078 PMC6785189 30975859

[11] Holmes SE (2019). Lower synaptic density is associated with depression severity and network alterations. Nat. Commun..

[12] Kang HJ (2012). Decreased expression of synapse-related genes and loss of synapses in major depressive disorder. Nat. Med..

[13] Miyamoto S, Leipzig JN, Lieberman JA, Duncan GE (2000). Effects of ketamine, MK-801, and amphetamine on regional brain 2-deoxyglucose uptake in freely moving mice. Neuropsychopharmacology.

[14] Chowdhury GM (2017). Transiently increased glutamate cycling in rat PFC is associated with rapid onset of antidepressant-like effects. Mol. Psychiatry.

[15] Kuhlman SJ (2013). A disinhibitory microcircuit initiates critical-period plasticity in the visual cortex. Nature.

[16] Sun Y (2016). Neuregulin-1/ErbB4 signaling regulates visual cortical plasticity. Neuron.

[17] Gu Y (2016). Neuregulin-dependent regulation of fast-spiking interneuron excitability controls the timing of the critical period. J. Neurosci..

[18] Yau HJ, Wang HF, Lai C, Liu FC (2003). Neural development of the neuregulin receptor ErbB4 in the cerebral cortex and the hippocampus: preferential expression by interneurons tangentially migrating from the ganglionic eminences. Cereb. Cortex.

[19] Vullhorst D (2009). Selective expression of ErbB4 in interneurons, but not pyramidal cells, of the rodent hippocampus. J. Neurosci..

[20] Neddens J, Buonanno A (2010). Selective populations of hippocampal interneurons express ErbB4 and their number and distribution is altered in ErbB4 knockout mice. Hippocampus.

[21] Fazzari P (2010). Control of cortical GABA circuitry development by Nrg1 and ErbB4 signalling. Nature.

[22] Wen L (2010). Neuregulin 1 regulates pyramidal neuron activity via ErbB4 in parvalbumin-positive interneurons. Proc. Natl Acad. Sci. USA.

[23] Tan GH (2011). Neuregulin 1 represses limbic epileptogenesis through ErbB4 in parvalbumin-expressing interneurons. Nat. Neurosci..

[24] Lu J, Tucciarone J, Lin Y, Huang ZJ (2014). Input-specific maturation of synaptic dynamics of parvalbumin interneurons in primary visual cortex. Proc. Natl Acad. Sci. USA.

[25] Grieco SF, Holmes TC, Xu X (2019). Neuregulin directed molecular mechanisms of visual cortical plasticity. J. Comp. Neurol..

[26] Grieco SF (2020). Subanesthetic ketamine reactivates adult cortical plasticity to restore vision from amblyopia. Curr Biol.

[27] Maeng S (2008). Cellular mechanisms underlying the antidepressant effects of ketamine: role of alpha-amino-3-hydroxy-5-methylisoxazole-4-propionic acid receptors. Biol. Psychiatry.

[28] Browne CA, Lucki I (2013). Antidepressant effects of ketamine: mechanisms underlying fast-acting novel antidepressants. Front Pharmacol..

[29] Abe Y, Namba H, Kato T, Iwakura Y, Nawa H (2011). Neuregulin-1 signals from the periphery regulate AMPA receptor sensitivity and expression in GABAergic interneurons in developing neocortex. J. Neurosci..

[30] Mahar I (2011). Subchronic peripheral neuregulin-1 increases ventral hippocampal neurogenesis and induces antidepressant-like effects. PLoS ONE.

[31] Long W (2003). Impaired differentiation and lactational failure of Erbb4-deficient mammary glands identify ERBB4 as an obligate mediator of STAT5. Development.

[32] Ghosh KK (2011). Miniaturized integration of a fluorescence microscope. Nat. Methods.

[33] Sun Y (2019). CA1-projecting subiculum neurons facilitate object-place learning. Nat. Neurosci..

[34] Pinto L, Dan Y (2015). Cell-type-specific activity in prefrontal cortex during goal-directed behavior. Neuron.

[35] Widman AJ, McMahon LL (2018). Disinhibition of CA1 pyramidal cells by low-dose ketamine and other antagonists with rapid antidepressant efficacy. Proc. Natl Acad. Sci. USA.

[36] Ali F (2020). Ketamine disinhibits dendrites and enhances calcium signals in prefrontal dendritic spines. Nat. Commun..

[37] Zhou P (2013). Interrogating translational efficiency and lineage-specific transcriptomes using ribosome affinity purification. Proc. Natl Acad. Sci. USA.

[38] Cohen S, Greenberg ME (2008). Communication between the synapse and the nucleus in neuronal development, plasticity, and disease. Annu. Rev. Cell Dev. Biol..

[39] Xu X (2016). Primary visual cortex shows laminar-specific and balanced circuit organization of excitatory and inhibitory synaptic connectivity. J. Physiol..

[40] Shi Y, Nenadic Z, Xu X (2010). Novel use of matched filtering for synaptic event detection and extraction. PLoS ONE.

[41] Shi L, Bergson CM (2020). Neuregulin 1: an intriguing therapeutic target for neurodevelopmental disorders. Transl. Psychiatry.

[42] Duman RS, Aghajanian GK (2012). Synaptic dysfunction in depression: potential therapeutic targets. Science.

[43] Beurel E, Song L, Jope RS (2011). Inhibition of glycogen synthase kinase-3 is necessary for the rapid antidepressant effect of ketamine in mice. Mol. Psychiatry.

[44] Zanos P, Thompson SM, Duman RS, Zarate CA, Gould TD (2018). Convergent mechanisms underlying rapid antidepressant action. CNS Drugs.

[45] Fogaca MV, Duman RS (2019). Cortical GABAergic dysfunction in stress and depression: new insights for therapeutic interventions. Front. Cell Neurosci..

[46] Gerhard DM (2020). GABA interneurons are the cellular trigger for ketamine’s rapid antidepressant actions. J. Clin. Investig..

[47] Mo A (2015). Epigenomic signatures of neuronal diversity in the mammalian brain. Neuron.

[48] Yang Y (2018). Ketamine blocks bursting in the lateral habenula to rapidly relieve depression. Nature.

[49] Hare BD, Duman RS (2020). Prefrontal cortex circuits in depression and anxiety: contribution of discrete neuronal populations and target regions. Mol. Psychiatry.

[50] Mahar I (2017). Disrupted hippocampal neuregulin-1/ErbB3 signaling and dentate gyrus granule cell alterations in suicide. Transl. Psychiatry.

[51] Bale TL (2019). The critical importance of basic animal research for neuropsychiatric disorders. Neuropsychopharmacology.

[52] Gorski JA (2002). Cortical excitatory neurons and glia, but not GABAergic neurons, are produced in the Emx1-expressing lineage. J. Neurosci..

[53] Cai DJ (2016). A shared neural ensemble links distinct contextual memories encoded close in time. Nature.

[54] Pnevmatikakis EA, Giovannucci A (2017). NoRMCorre: an online algorithm for piecewise rigid motion correction of calcium imaging data. J. Neurosci. Methods.

[55] Zhou, P. et al. Efficient and accurate extraction of in vivo calcium signals from microendoscopic video data. *Elife* **7**, 10.7554/eLife.28728 (2018). 10.7554/eLife.28728 PMC5871355 29469809

[56] Pnevmatikakis EA (2016). Simultaneous denoising, deconvolution, and demixing of calcium imaging data. Neuron.

[57] Friedrich J, Zhou P, Paninski L (2017). Fast online deconvolution of calcium imaging data. PLoS Comput Biol..

[58] Johnston, K. G. et al. Robust population single neuronal calcium signal extraction using SCOUT allows for longitudinal analysis of behavior-associated neural ensemble dynamics. *bioRxiv*10.1101/2020.08.26.268151 (2020).

[59] Burgess A (2010). Loss of human Greatwall results in G2 arrest and multiple mitotic defects due to deregulation of the cyclin B-Cdc2/PP2A balance. Proc. Natl Acad. Sci. USA.

[60] Xu X, Olivas ND, Levi R, Ikrar T, Nenadic Z (2010). High precision and fast functional mapping of cortical circuitry through a combination of voltage sensitive dye imaging and laser scanning photostimulation. J. Neurophysiol..

[61] Sun Y (2014). Cell-type-specific circuit connectivity of hippocampal CA1 revealed through Cre-dependent rabies tracing. Cell Rep..

[62] Xu X, Olivas ND, Levi R, Ikrar T, Nenadic Z (2010). High precision and fast functional mapping of cortical circuitry through a novel combination of voltage sensitive dye imaging and laser scanning photostimulation. J. Neurophysiol..

[63] Shepherd GM, Pologruto TA, Svoboda K (2003). Circuit analysis of experience-dependent plasticity in the developing rat barrel cortex. Neuron.

[64] Fisher R (1918). The correlation between relatives on the supposition of Mendelian inheritance. Trans. R. Soc. Edinb..

[65] Laird N, Ware JH (1982). Random-effects models for longitudinal data. Biometrics. Int. Biom. Soc..

[66] Henderson, C. R. Sire evaluation and genetic trends. *J Anim Sci.* **1973**, 10–41, 10.1093/ansci/1973.Symposium.10 (1973).

[67] McLean, R. A., Sanders, W. L. & Stroup, W. W. A Unified approach to mixed linear models. *Am Stat.* **45**, 54–64 (1991).

[68] Indersmitten T (2019). In vivo calcium imaging reveals that cortisol treatment reduces the number of place cells in Thy1-GCaMP6f transgenic mice. Front. Neurosci..

[69] Stobart JL (2018). Long-term in vivo calcium imaging of astrocytes reveals distinct cellular compartment responses to sensory stimulation. Cereb. Cortex.

[70] Stobart JL (2018). Cortical circuit activity evokes rapid astrocyte calcium signals on a similar timescale to neurons. Neuron.

[71] Livak KJ, Schmittgen TD (2001). Analysis of relative gene expression data using real-time quantitative PCR and the 2(-Delta Delta C(T)) method. Methods.

