# Supplementary material for: Neuregulin signaling mediates the acute and sustained antidepressant effects of subanesthetic ketamine
Source: Transl Psychiatry. 2021 Feb 24;11:144. doi: 10.1038/s41398-021-01255-4 (PMC7904825; doi:10.1038/s41398-021-01255-4)
Supplement: Supplementary file 1 — Supplementary Table 1 [file 41398_2021_1255_MOESM1_ESM.pdf]

Supplementary Table 1. Experiments, mouse strains and relevant procedures

▪Forced Swim Test (Figure 1)

**Mice Used**	**Treatments**	**Experiments**
C57BL/6 (N=134)	Saline (s.c),<br>ketamine (10mg/kg; s.c.),<br>HNK (10mg/kg; s.c.),<br>NRG1 (1µg; s.c.),<br>PD158780 (10mg/kg; s.c.)	Forced Swim Test Behavior
PV-Cre; ErbB4$^{fl/fl}$ (N=18)	Saline (s.c) or ketamine (10mg/kg; s.c.)	Forced Swim Test Behavior

▪Miniscope recording (Figure 2)

**Mice Used**	**Treatments**	**Experiments**
C57BL/6 (N=12)	Saline (s.c) or ketamine (10mg/kg; s.c.)	miniscope recording of mPFC in freely behaving animals

▪IPSC Recording from excitatory pyramidal cells (PYRs) (Figure 3)

**Mice Used**	**Treatments**	**Experiments**
C57BL/6 (N=5)	Bath ketamine treatment	PYR IPSC recording after acute bath application of ketamine to cortical slices
C57BL/6 (N=5)	MK-801 treatment	PYR IPSC recording after MK-801 injection
C57BL/6 (N=5)	HNK treatment	PYR IPSC recording 24 hr after HNK injection
C57BL/6 (N=28)	Saline (s.c) or ketamine (10mg/kg; s.c.)(1, 24, 48, 72 h, 1 wk)	PYR IPSC recording at different times after ketamine injection

▪NRG1 and ErbB4 mRNA Quantification (Figure 4)

**Mice Used**	**Treatments**	**Experiments**
PV-Cre; fsTRAP (N=3)	none	Perfusion and characterization of EGFP expression in PV cells
Emx1-Cre; fsTRAP (N=3)	none	Perfusion and characterization of EGFP expression in excitatory cells
PV-Cre; fsTRAP (N=125)	Saline (s.c) or ketamine (10mg/kg; s.c.) (24, 48, 72 h, 1 wk)	Quantification of PV-specific NRG1 and ErbB4 expression by qPCR
Emx1-Cre; fsTRAP (N=50)	Saline (s.c) or ketamine (10mg/kg; s.c.)(24, 48, 72 h, 1 wk)	Quantification of excitatory-specific NRG1 and ErbB4 expression by qPCR

▪pCREB Quantification (Figure 4)

Mice Used	Treatments	Experiments
PV-Cre; Ai9 (N=30)	Saline (s.c) or ketamine (10mg/kg; s.c.)(24, 48, 72 h, 1 wk)	Perfusion and measurement of pCREB immunostaining

▪EPSC Recordings from PVs using LSPS (Figure 4; Supplementary Figure 3)

Mice Used	Treatments	Experiments
PV-Cre; Ai9 (N=32)	Saline (s.c) or ketamine (10mg/kg; s.c.)(24, 48, 72 h, 1 wk)	PV EPSC recording at different times after ketamine injection

* Please refer to the main text and Methods for more detailed information.
