# Supplementary figures and images for: Neuregulin signaling mediates the acute and sustained antidepressant effects of subanesthetic ketamine
Source: Transl Psychiatry. 2021 Feb 24;11:144. doi: 10.1038/s41398-021-01255-4 (PMC7904825; doi:10.1038/s41398-021-01255-4)

**A**

### Spontaneous IPSC Amplitudes

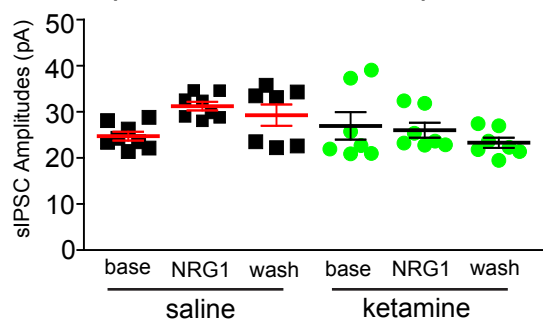**B**

### Spontaneous IPSC Frequencies

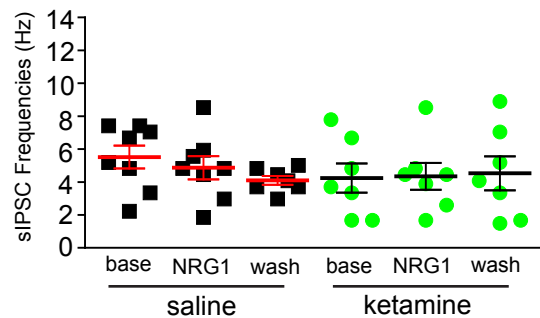**C**

### Paired-Pulse Ratios

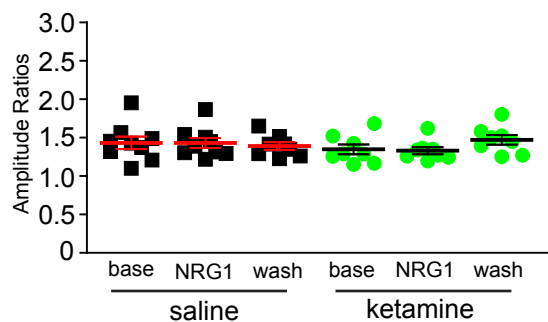

Supplement: Supplementary file 2 — Supplementary Figure 1 [file 41398_2021_1255_MOESM2_ESM.pdf]

**A**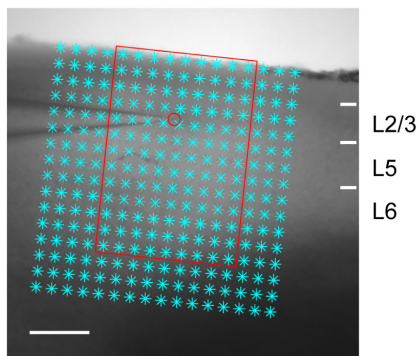**B**

72 h post-Ket PV cells

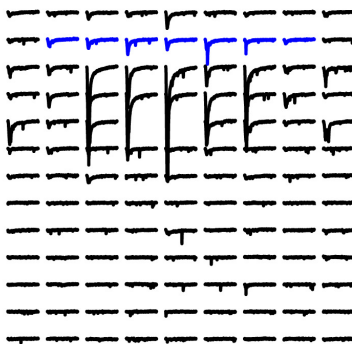**C**

72 h post-Ket + NRG1

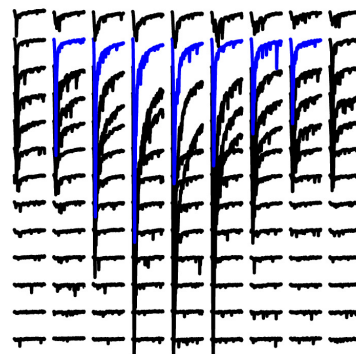**D**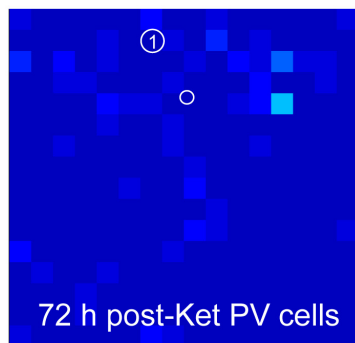**E**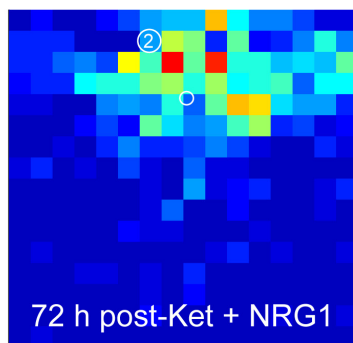**F**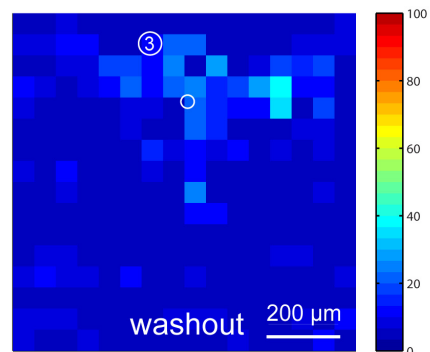

①

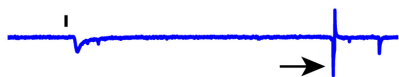

②

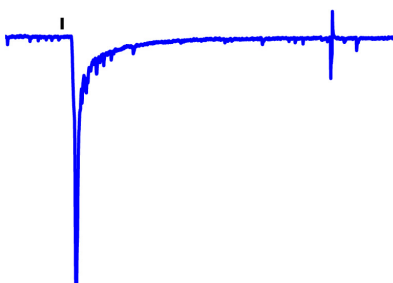

③

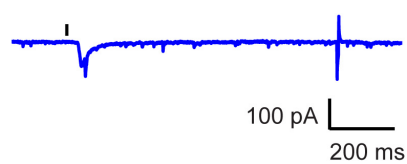

Supplement: Supplementary file 3 — Supplementary Figure 2 [file 41398_2021_1255_MOESM3_ESM.pdf]

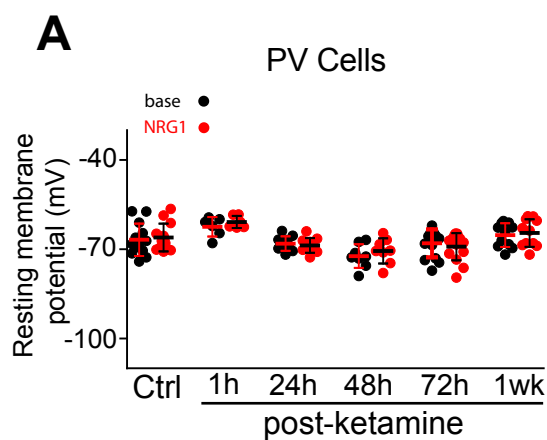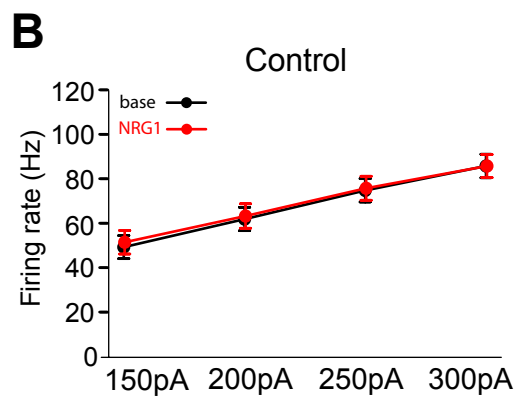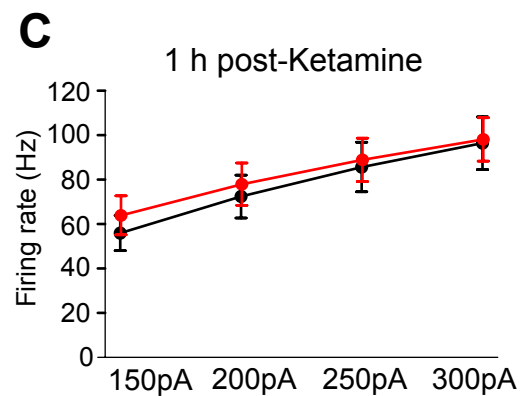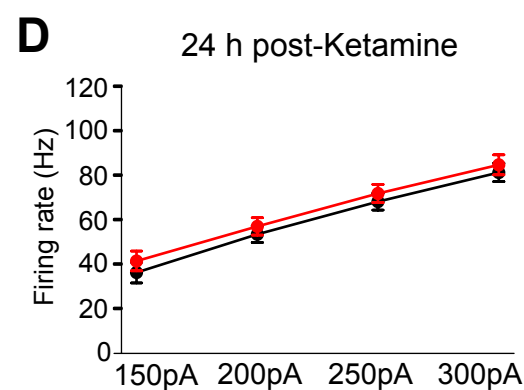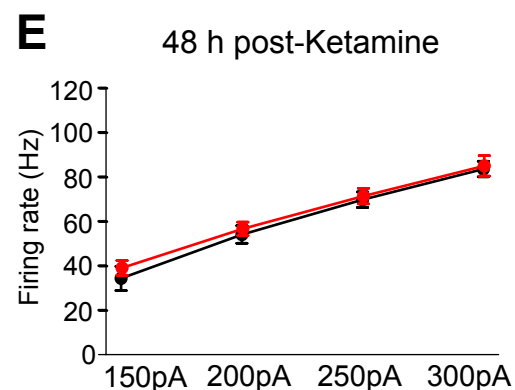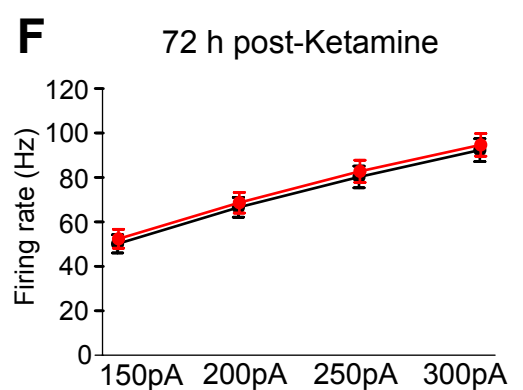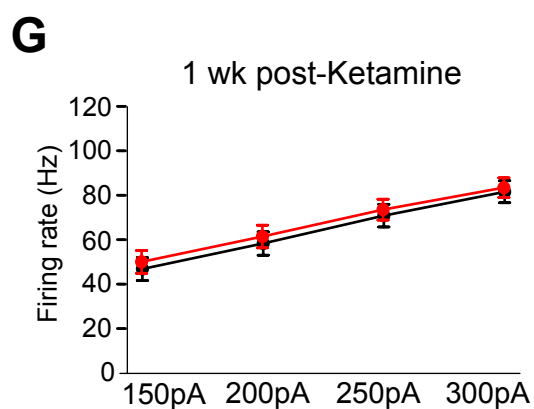

Supplement: Supplementary file 4 — Supplementary Figure 3 [file 41398_2021_1255_MOESM4_ESM.pdf]

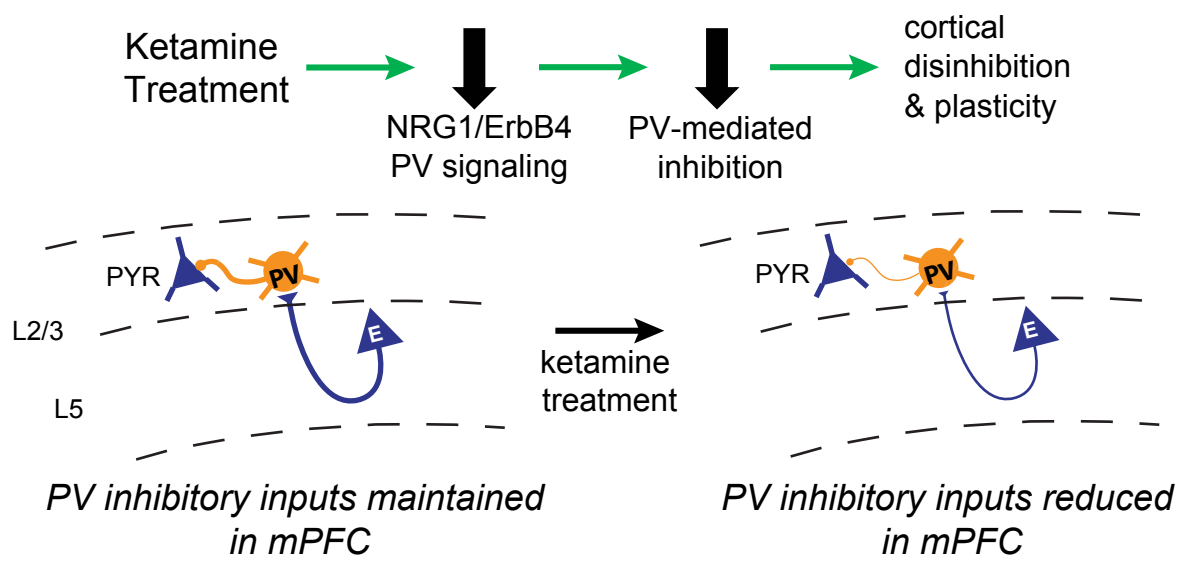

Supplement: Supplementary file 5 — Supplementary Figure 4 [file 41398_2021_1255_MOESM5_ESM.pdf]
