# Supplementary material for: Neuregulin signaling mediates the acute and sustained antidepressant effects of subanesthetic ketamine
Source: Transl Psychiatry. 2021 Feb 24;11:144. doi: 10.1038/s41398-021-01255-4 (PMC7904825; doi:10.1038/s41398-021-01255-4)
Supplement: Supplementary file 6 — Supplementary Figure Legends [file 41398_2021_1255_MOESM6_ESM.docx]

**Supplementary Figure 1.** Ketamine treatment does not have a significant effect on spontaneous IPSCs or paired-pulse ratios in recorded excitatory neurons. (A) Spontaneous IPSC amplitudes in L2/3 pyramidal neurons from mPFC cortical slices from animals treated with saline (n=7 cells) or ketamine (10 mg/kg; s.c.) (n=7 cells) 24 hours after treatment. (B) Spontaneous IPSC frequencies in L2/3 pyramidal neurons from mPFC cortical slices from animals treated with saline (n=8 cells) or ketamine (10 mg/kg; s.c.) (n=7 cells) 24 hours after treatment. (C) Paired-pulse ratios of IPSC amplitudes in L2/3 pyramidal neurons in mPFC slices after L5 electrical stimulation pulses. Animals were treated with saline (n=8 cells) or ketamine (10 mg/kg; s.c.) (n=7 cells) and recordings were made 24 hours later.

**Supplementary Figure 2.** Laser scanning photostimulation (LSPS). LSPS allows for quantitative mapping of local excitatory synaptic inputs to recorded PV neurons from laminar circuits in a relatively large cortical region. (A-F) recordings maintained on the same neuron during baseline, treatment, and washout. (A) PV/fast-spiking (FS) cells are targeted by tdTomato expression in PV-Cre; Ai9 mouse mPFC slices. A mPFC slice image superimposed with photostimulation sites (cyan circles) spaced at 60 µm x 60 µm. The red circle indicates the tip of a recording electrode and the cell body location of a recorded L2/3 PV interneuron from a mouse treated with ketamine. Scale bar = 200 µm. (B-C) The plot of excitatory postsynaptic current (EPSC) responses from the recorded PV cell at the selected sites within the region shown by the red rectangle in (A) before and after bath application of exogenous recombinant NRG1 in a mPFC slice from a mouse treated with ketamine. (D-F) Representative example of bath NRG1 enhancement of excitatory synaptic inputs to a ketamine-treated PV cell. Quantitative input maps were obtained before (D), during (E) and after washout (F) of bath applied NRG1. The spatial scale in (F) indicates 200 µm. The color scale (F) indicates average integrated input strength at individual map sites. The warmer color indicates stronger input strength. The small white circle indicates the cell body location of the ketamine-treated PV neuron. Each map site (color pixel) is spaced at 60 µm x 60 µm. (Below D-F) Synaptic input responses at the specified, numbered sites. The response traces are plotted for 1200 ms, with 200 ms baselines before a 1ms photostimulation (black ticks above the traces). Current injection responses (5pA, 5 ms; pointed by the arrow) allow for the monitoring of access resistance during the mapping experiment. Any experiment in which the access resistance changes by >20% during the course of the experiment were excluded from the analysis.

**Supplementary Figure 3.** Resting membrane potentials, intrinsic membrane excitability and firing rates of PV neurons do not change after ketamine treatment and with NRG1 bath application. (A,G) Recordings are from different neurons and different mice at time points that are on different days (Control, 1, 24, 48, 72 hours, 1 week) after treatment, and recordings are maintained on the same neuron during baseline then NRG1 treatment. (A) Resting membrane potentials of PV cells of control, and 1, 24, 48, 72 hours and 1 week after ketamine treatment do not differ, and they are not changed by bath application of exogenous recombinant NRG1. (B-G) Plots of the overall relationship between PV cell firing rates and current injection strengths at 1 hours (C), 24 hours (D), 48 hours (E), 72 hours (F) or 1 week (G) post-ketamine treatment as compared to controls (B). The data values are represented as means ± SEM and at each condition [controls (n = 10 cells), 1h (n = 6), 24h (n = 8), 48h (n = 8), 72h (n = 9) and 1wk (n = 11)]. Resting membrane potentials and firing rates were determined before and after bath application of NRG1 (A-G).

**Supplementary Figure 4.** A molecular, cellular and circuit model to account for ketamine-mediated antidepressant effects. A model of the effect of ketamine of mPFC PV interneurons circuitry and NRG1/ErbB4 signaling. A subanesthetic ketamine treatment reduces NRG1/ErbB4 signaling in PV interneurons. We do not yet understand the detailed molecular mechanisms for how/why NRG1 down-regulation in PV interneurons occurs after ketamine administration. This is a topic for future investigation. This results in a decrease of excitatory inputs onto PV interneurons. As a result of this reduction of input, PV interneurons in turn reduce firing activities. Therefore, L2/3 excitatory neurons receive less PV inhibitory input, resulting in disinhibited cortical function and increased excitatory neuron activity. These conditions modulate cortical circuit plasticity to counteract depression-like behaviors.
